# Stretchable Laminates with Tunable Structural Colors from Layered Stacks of Elastomeric, Ionic, and Natural Polymers

**DOI:** 10.1021/acsami.5c01880

**Published:** 2025-03-31

**Authors:** Yiming Zhang, Paraskevi Flouda, Valeriia Poliukhova, Alexandr V. Stryutsky, Valery V. Shevchenko, Vladimir V. Tsukruk

**Affiliations:** † School of Materials Science and Engineering, Georgia Institute of Technology, Atlanta, Georgia 30332, United States; ‡ Department of Chemical and Environmental Engineering, University of Arizona, Tucson, Arizona 85721, United States; § Institute of Macromolecular Chemistry of the National Academy of Sciences of Ukraine, Kyiv 02155, Ukraine

**Keywords:** cellulose nanocrystals, branched ionic polymers, stretchable laminated composites, humidity and heat responsive colors

## Abstract

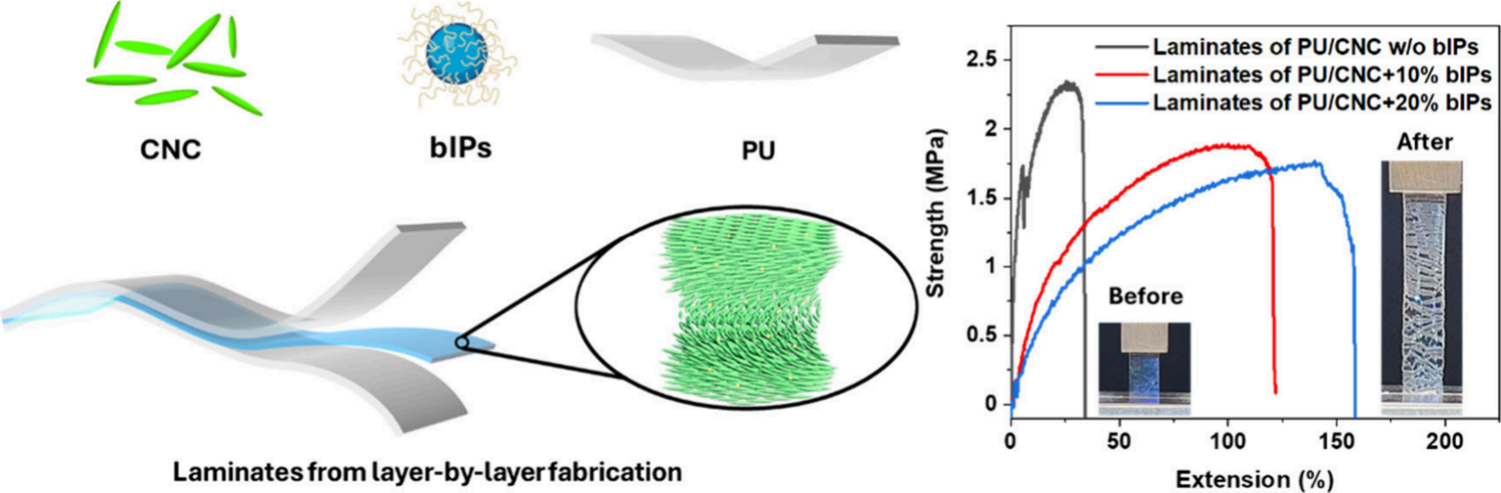

Natural polymers such as plant-derived cellulose nanocrystals (CNCs) are renowned for color iridescence due to their internal helical organization, but they show modest stretchability and bending abilities, because of the brittle nature of highly crystalline needlelike nanocrystals. Herein, we report the highly stretchable composite materials built from these nanocrystals and branched ionic polymers (bIPs) with terminal amine-terminated poly(*N*-isopropylacrylamide) (PNIPAM) stacked between elastomeric layers. These layered elastomeric composites preserve the high mechanical stretchability of polyurethane outer layers up to 150%. Furthermore, the toughness increased manyfold, due to the sequential initiation and arresting of concurrent transversal cracks within the reinforcing central nanocomposite layer. Moreover, vivid structural colors of CNC helical organization preserved within these laminated composites show the ability to respond to humidity and temperature. We suggest that these elastomeric composite laminates with preserved structural colors of helical nanocellulose organization can be considered to be promising candidates for demanding applications such as robust wearable sensors, flexible optical labels, and photonic devices.

## Introduction

Cellulose nanocrystals (CNCs) are well-known plant-derived needlelike nanomaterials with intriguing structural colors and potential for highly mechanically performing materials. Upon slow evaporation, CNC suspension self-assembles into a left-handed chiral nematic liquid-crystal phase with helical organization, following formation of solid films exhibiting unique color iridescence. This special optical property, combined with the abundant availability of cellulose—the primary building block of plant cell walls in nature—position CNCs as promising candidates for environmentally friendly pigments, biophotonics, chiroptical sensing, and prospective optoelectronic devices.^1,2^ However, unlike traditional biomacromolecules, CNCs are rigid rodlike nanoneedles with a high aspect ratio, which form bundles and helicoidal organization stabilized by interparticle interactions (mainly Coulombic forces and hydrogen bonding).^3^ These interactions govern their long-range aggregations but also result in fragile assemblies of rigid nanoneedles with limited dissipation of fracture energy, leading to excessive brittleness and cracking in conventional CNC films. Adding fillers such as polymers, biomaterials, nanoparticles, ionic, and hydrogel components during the evaporation-induced self-assembly (EISA) can improve mechanical performance resulting in enhanced flexibility of CNC materials,^4−6^ but preserving controllable color appearance remains challenging.^7,8^ To improve flexibility and stretchability, other nanocellulosic materials such as cellulose nanofibers (CNFs) or polysaccharides, have been explored for improved flexibility but iridescent structural colors are usually compromised.^5,9,41^

To date, the incorporation of additional components into CNC matrices has a critical range limit of 10–40 wt % to preserve the chiral organization and maintain the vivid structural color of CNCs within the visible spectrum.^5^ However, this limited loading is insufficient to achieve the desired flexibility and stretchability needed to withstand larger deformations. Therefore, alternative approaches have been explored to improve stretchability without compromising structural colors. For instance, MacLachlan et al. impregnated helical CNC organizations into elastomers by swelling CNC films with acrylate monomers followed by post-polymerization.^10^ Ge et al. induced post-cross-linking in the mixture of CNC/poly(ethylene glycol) dimethyl acrylate (PEGDMA) to acquire enhanced stretchability and mechanochromic properties without compromising the structural integrity and colors.^11^ Additionally, layer-by-layer fabrication have been investigated to create multilayered structures that bridge the softness of elastomers with the multifunctionality of nanomaterials.^12^ By carefully controlling interlayer interactions, it is possible to form in-situ double networks that support the low-dimensional materials and prevent early catastrophic failure, thus achieving flexibility and mechanical robustness. For instance, Rui et al. reported the alternate stacking of CNCs and CNFs that resulted in strong chiral optical response with enhanced flexibility.^13^

Furthermore, responsive photonic materials have garnered increasing interest as CNCs demonstrate the ability to switch color by expanding/retracting its helical pitch length upon external stimuli. Responsive chiral CNC films with detectable color shifts can be achieved by adding hydrophilic polymers or hygroscopic molecules such as poly(ethylene glycol), poly(acrylamide), or glycerol^14,15^ or by incorporating thermoresponsive polymers such as PNIPAM, which undergoes dramatic shape changes upon reaching the lower critical solution temperature (LCST).^16^ For instance, Sun et al. reported significant blue shifts up to 87 nm for CNC/PNIPAM composite films upon heating at 40 °C.^17^ In another example, branched ionic polymers with weakly tethered macro-cation arms exhibit high mobility of the terminal PNIPAM chains, because of their ability to desorb and dynamically hop between different core sites.^18,26,34^ Upon reaching the LCST, this dynamic behavior triggers morphological reconfiguration, leading to micelle aggregation into larger multimolecular associations.^18^

**Scheme 1:**
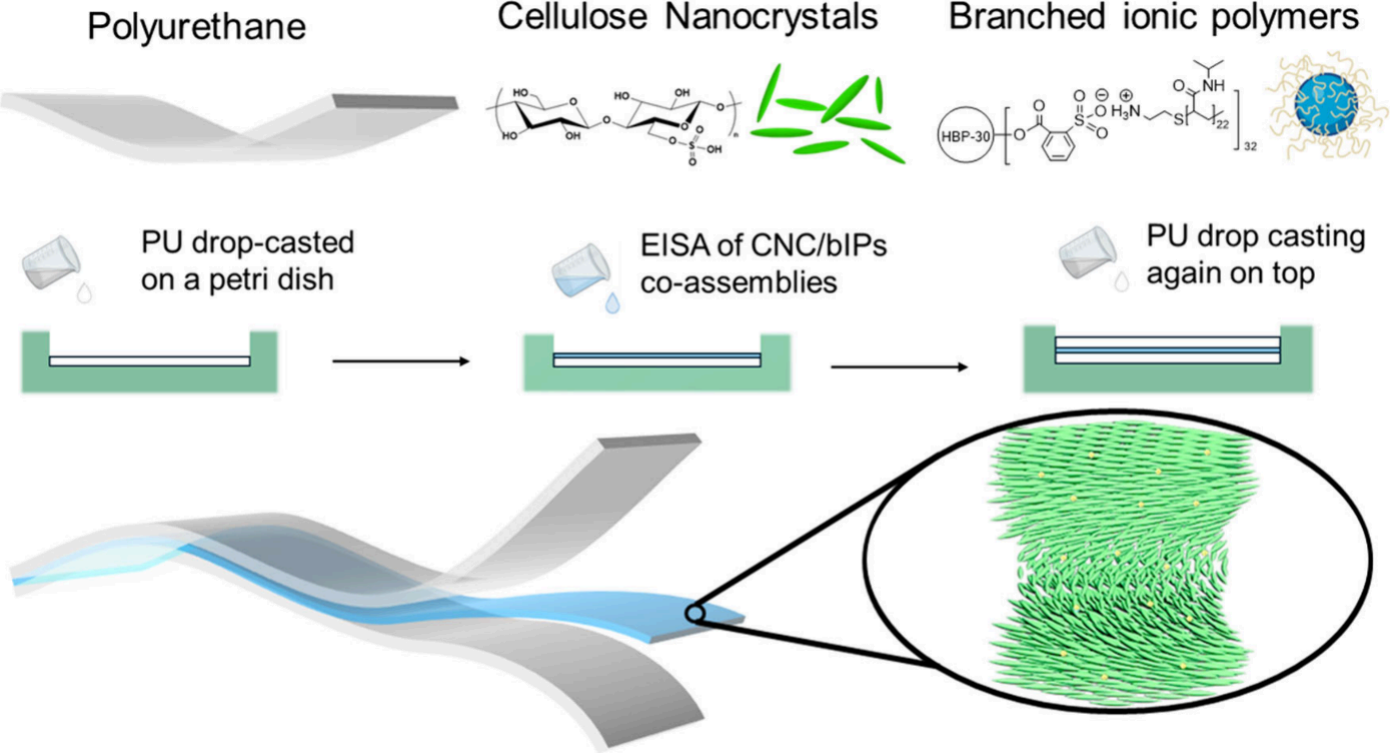
Schematic Representation and Chemical Structures of the Components (Top) and Step-by-Step Assembly Method (Middle) Including the Self-Assembly of Cellulose Nanocrystals and Ionic Polymers within Central Nanocomposite Layer to Create Integrated Elastomeric Laminates by the Stacking of the Different Layers (Bottom)

Here, we employed a step-by-step assembly method to create stretchable and structurally colored laminates where iridescent CNC layers mediated by thermoresponsive branched ionic polymers (bIPs) were stacked between polyurethane (PU) elastomeric layers (Scheme 1). This approach enabled the combination of structural color enhancement in the central CNC layer, driven by the presence of ionic polymer components, with improved mechanical performance provided by supporting elastomeric layers. The resulted iridescent elastomeric laminates possess large stretchability of up to 150% and demonstrate bright colors and induced color variability with large redshift by 50–100 nm upon increased humidity and temperature. The layered composite design takes advantage of the flexibility of elastomeric layers to enhance mechanical performance, further reinforced by the ionic coupling between bIPs and CNCs. This interaction helps mediate crack initiation and propagation while preserving the color iridescence up to high strains.

## Materials and Methods

### Synthesis of Branched Ionic Polymers

Poly(*N*-isopropylacrylamide) amine terminated (PNIPAM, *M*_n_ = 2500 g/mol) and 2-sulfobenzoic acid cyclic anhydride (≥95%) were purchased from Sigma–Aldrich and used as received. Hyperbranched aliphatic polyester polyol (HbP–OH, BoltornH30 from Perstorp, with molecular weight *M*_w_ = 3500 g/mol bearing 32 hydroxyl terminal groups) was purified before use. Ultrapure water (resistivity ≥ 18.2 MΩ cm) was obtained from a three-stage Millipore Milli-Q Plus 185 purification system and used for all the experiments. (See more details in the Supporting Information.)

Synthesis of the branched thermoresponsive ionic polymers was conducted based on our previous work,^19,26^ by exhaustive acylation of hyperbranched polyol cores (HbP–OH) with 2-sulfobenzoic acid cyclic anhydride, followed by the neutralization of the sulfonate groups with the amine groups of PNIPAM (see more details in the Supporting Information). Due to the ionizable end groups, the synthesized polymers belong to a category of polyelectrolyte macromolecules with branched architectures.^20^ The synthesized bIPs were dissolved in water at a concentration of 10 mg/mL with stirring at room temperature.

### Preparation of Cellulose Nanocrystals

The preparation of CNC dispersion was conducted using sulfuric acid hydrolysis, a well-established method.^21^ Generally, the softwood pulp (bleached kraft pulp, International Paper) was first rinsed with deionized (DI) water and then torn apart into smaller pieces. After air drying, 34 g of dry pulp was added to sulfuric acid (600 mL) at a concentration of 64% at 45 °C for hydrolysis. After 60 min of hydrolysis, the obtained suspension was diluted 10 times with Nanopure water to terminate the reaction. After the mixture was allowed to sit overnight for sedimentation, the clear supernatant was discarded, and the remaining white precipitate was collected and purified twice by centrifugation at 6000 rpm for 5 min.

The obtained white suspension was then redispersed in Nanopure water and placed inside the dialysis membrane tube with 14 kDa cutoff for dialysis against water for several days, during which the water was changed every 3 h until the pH value of the water was consistent. After dialysis, the white suspension was centrifuged at 12 000 rpm for 20 min, and the clear supernatant was collected. The final CNC suspension was ultrasonicated for 4 min 30 s at 50% amplitude with 5 s on/5 s off using a tip sonicator (Qsonica Model Q125, 700 W). The as-prepared CNC dispersion was cast and dried in a Petri dish to determine the final concentration, which was 0.74 wt %.

### Fabrication of Multilayered Laminates

The laminated films were prepared by the alternate stacking of PU (Alberdingk Boley, U 3200, used after drying) and a mixture of CNC and bIP by the drop-casting method. Generally, PU dispersion (2 mL, redispersed in DCM in 40 mg/mL) was drop-casted onto a glass Petri dish (30 mm diameter × 15 mm) to dry in the ambient environment, producing one layer of transparent PU film. Separately, varying amounts of the bIPs solution (10 mg/mL) were added to the CNC dispersion (2 mL) under stirring. The mixtures were further sonicated for 5 min and then drop-casted onto the preformed PU films. This step was followed by evaporation-induced self-assembly at the ambient environment to yield a second layer of the multilayered films.^22^ Finally, another 2 mL of PU dispersion was drop-cast on the CNC/bIP layer and dried in the ambient environment for another hour. Lastly, the obtained films were carefully peeled off the Petri dish to obtain freestanding composite films of three layers for further characterization.

### Characterization

Attenuated total reflection Fourier transform infrared (ATR–FTIR) spectra were collected using a Bruker Vertex 70 FTIR spectrometer within 4000 to 1200 cm^–1^ at a resolution of 2 cm^–1^. The proton nuclear magnetic resonance (^1^H NMR) spectrum of the bIPs was recorded with a Varian VXR-400 MHz spectrometer with DMSO-*d*_6_ as the solvent to confirm its chemical structure.

Thermal properties were studied by thermogravimetric analysis (TGA) and differential scanning calorimetry (DSC) using Q50 (TA Instruments, USA) and Q2000 (TA Instruments) equipment, respectively. Samples were dried in a vacuum at 80 °C before thermal testing. TGA was performed within the temperature range of 20–700 °C at a heating rate of 20 °C/min in the air atmosphere. DSC was conducted within the temperature range of −80 to 150 °C at a heating/cooling rate of 20 °C/min in an air atmosphere. Two heating/cooling cycles were conducted, and the curve from the second cycle was used to determine a glass-transition temperature (*T*_g_). A Zetasizer Nano ZS (Malvern Instrument) was used to measure the hydrodynamic radius and zeta potential for solutions in polystyrene cuvettes at different temperatures. The CNCs and bIPs mixture was measured at its original concentration, while the bIP solution was diluted 10-fold to approximate its actual concentration within the mixture. For measurement at elevated temperatures, samples were first equilibrated in the oven at the set temperature for 5 min and then being quickly transferred and measured to ensure uniform heating and prevent temperature gradient. The results were averaged over three individual measurements, and additional long-time nanoparticle size evolution was monitored from several days up to a week after suspension preparation. No significant changes in particle sizes were observed within the observation time.

Optical microscopy was conducted on an Olympus optical microscope equipped with polarizers in dark-field mode. UV–vis spectroscopy was performed by using a Shimazu Model UV-3600 Plus UV–vis-NIR spectrophotometer with a scanning range of 250–700 nm to determine the reflectance peak of the films. For humidity/thermoresponsive testing, the samples were first cut into identical strips (10 mm × 3 mm) and then conditioned in the incubator with a humidifier/vaporizer to create the variant humidity and temperature conditions, monitored by a hygrometer, before putting in a capped quartz cuvette and measuring the UV–vis reflectance peak to characterize the responsive behavior. All measurements were repeated three times for film samples to be averaged. Circular dichroism (CD) measurement was performed on an AP Chirascan-plus CD spectrometer with the films mounted on the sample holder.

The morphology of materials was characterized using atomic force microscopy (AFM) with a Bruker Dimension Icon microscope operated in a normal tapping mode in air with a resolution of 512 × 512 pixels at the scan rate of 0.5 Hz using the AFM probe (HQ:XSC11/AL BS) with a spring constant of 1.1–5.6 N/m and a nominal tip radius of 8 nm, according to the established procedure.^23^ Nanoscope Analysis 2.0 (Bruker) was used for image analysis. To probe the local morphology, CNC suspension and their mixtures with bIPs, as well as the pure bIP solution, were drop-cast onto fresh piranha-treated silicon wafers.

Cross-sectional images of fractured films were captured by scanning electron microscopy (SEM) Hitachi SU8210 instruments at an accelerating voltage of 3.0 kV. The SEM cross sections were obtained by cracking the film in liquid nitrogen and adhering the pieces onto carbon tape attached to the cross-sectional SEM stub. Samples were sputter coated with a 15-nm gold–platinum layer before SEM imaging.

Tensile tests were performed on a Mark-10 tensile tester at a uniaxial stretching speed of 30 mm min^–1^. Before the test, samples were cut into a rectangular shape in 3 mm × 10 mm dimensions. At least four tests were conducted for samples in different bIP loading levels. A paper stamp template was utilized to help stabilize the sample during mounting for tensile tests to collect full stress–strain curves. Videos were taken to capture the whole stretching process of the tests including the initial, cracking, and breaking stages to compare the failure behavior of the samples.

## Results and Discussion

### Hyperbranched Ionic Polymers (bIPs)

**Figure 1:**
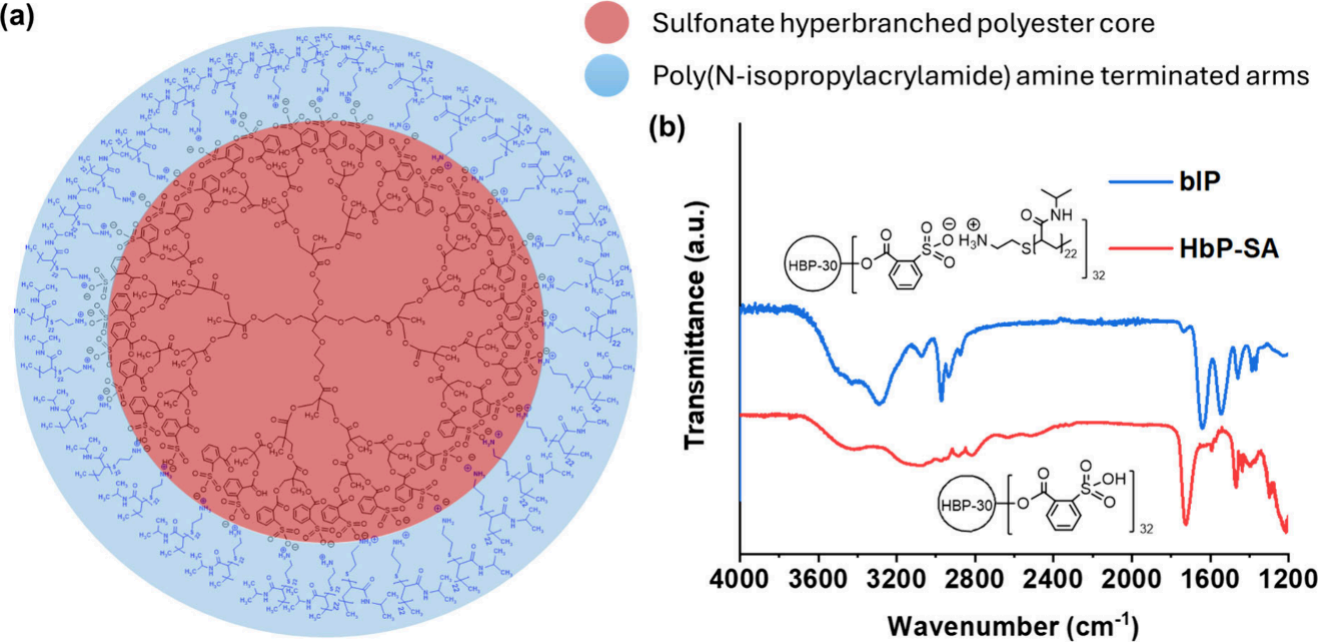
(a) Schematic representation of the branched ionic polymer where the red circle indicates the sulfonate polyester core while PNIPAM macrocation arms are highlighted in blue. (b) FTIR spectra of the oligomeric hyperbranched sulfonate acid (HbP-SA) and the bIP compound with 32 hydrophilic PNIPAM amine terminated macrocations.

The synthesized bIPs are protic anionic hyperbranched ionomers with 32 terminal sulfonate primary ammonium ionic groups with terminal chains of PNIPAM macrocations (see Figure 1a, as well as Figure S1 in the Supporting Information).^39^ The difference in the p*K*_a_ values of the sulfonic acid groups and an acid conjugated to the primary amino groups of the PNIPAM is 13.3, according to ACD/Laboratory calculations, suggesting a complete proton transfer from the acid to the base.^24^ In addition, the protic ionic groups and thermoresponsive PNIPAM arms at temperatures below LCST (32 °C) contribute to hydrogen and electrostatic bonding of the bIP with hydrophilic aliphatic hydroxyl and sulfonic acid groups of the CNCs.

The synthesis of the bIPs is based on exhaustive acylation of hydroxyl groups of the HbP–OH with 2-sulfobenzoic acid cyclic anhydride followed by neutralization of the reaction product with primary aliphatic amino end-groups of PNIPAM (Figure S1 in the Supporting Information). The chemical structure of the synthesized compound is confirmed by FTIR (Figure 1b) and ^1^H NMR spectroscopy (Figure S2 in the Supporting Information).

Peak sharpening and shifting could be found when amine-terminated PNIPAM was introduced to prepare bIPs (Figure 1b). First, sharper peaks were observed in 4000–3000 cm^–1^ due to the formation of ionic interactions between sulfonic acid and amine groups.^25^ Then, the absorption of amide I (*v*C=O) and amide II (δN–H) from NH_2_–PNIPAM was also observed at ∼1642 cm^–1^ and ∼1545 cm^–1^, respectively.^26,27^ Next, *v*C–H from CH_2_ at 2800–3000 cm^–1^ is intensified due to the presence of PNIPAM chains, with the onset of *v*N–H from NH_2_ from amine at ∼3288 cm^–1^ (detailed interpretation in the Supporting Information).^18^

The ^1^H NMR spectrum shows signals of protons of methyl (0.80–1.16 ppm) and methylene (1.19, 1.45, 2.14–4.50 ppm) groups of hyperbranched oligoester cores and PNIPAM arms, signals of protons attached to tertiary carbon atoms of PNIPAM fragments (1.97, 3.84 ppm) and signals of protons of aromatic rings, amide groups (6.97–8.04 ppm) and primary ammonium cations (8.39 ppm) as shown in Figure S2.^28^ The molecular structure is confirmed by both positions of signals corresponding to characteristic groups and ratios of their areas (detailed analysis in the Supporting Information).

Finally, the molecular weight (MW) for the bIP was determined as 90 326 g/mol based on the MW of HBP-SA from the acid–base titration technique (details in the Experimental Section and the Supporting Information) and terminal groups MW of PNIPAM (Table 1). This value is close to the theoretical value of 89 670 g/mol based on the targeted chemical structure and on the similar architectures by PNIPAM macrocations in our previous studies.^18,26,36^

**Table 1:** Summary of Molecular Weight (MW), Glass-Transition Temperature (*T*_g_), and Thermal Decomposition Temperature (*T*_d_) of the bIP Exploited Here

sample	calculated MW (g/mol)	found MW (g/mol)	*T*_g_ (°C)	*T*_d_ (°C)
bIP with 32 PNIPAM tails	89670	90326	94	308

### Thermal Behavior

The glass-transition temperature, *T*_g_, for the bIP is 94 °C which is significantly lower in comparison with linear PNIPAM counterparts (110–140 °C) (see Table 1, as well as Figure S3).^29,30^ That is due to the influence of the lower molecular weight of ester hyperbranched core (estimated MW = 9632 g/mol).^19,26^ The *T*_g_ values of compounds similar to the initial polyetherpolyol are 25–40 °C.^31,32^ According to the TGA (see Table 1, as well as Figure S4), the synthesized compound is characterized by a temperature of thermo-oxidative degradation (*T*_d_) of 308 °C with the onset of degradation at 100 °C.

**Figure 2:**
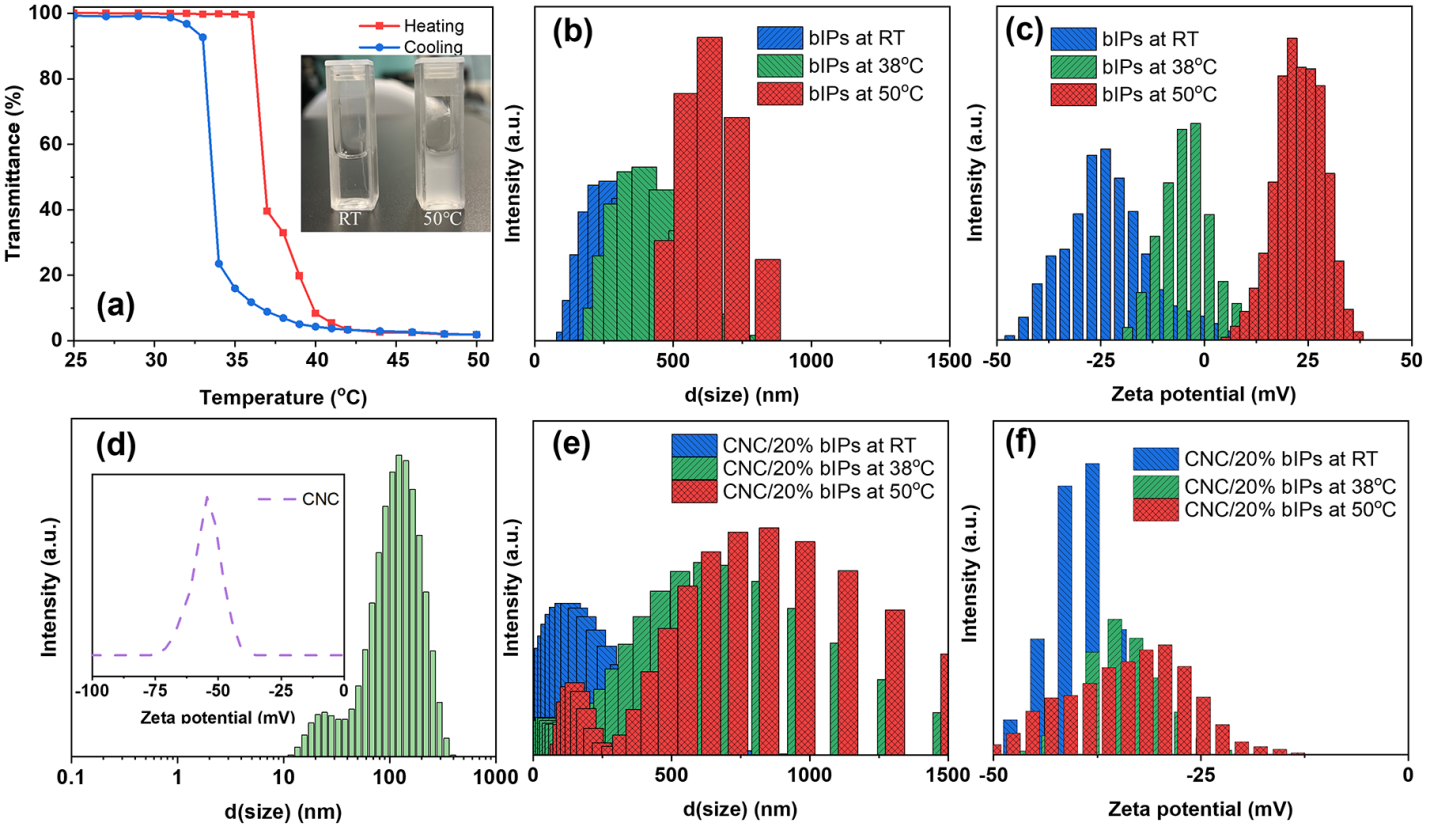
(a) Transmittance, (b–d) size, and (e, f) zeta potential of the bIP, CNC suspension, and their mixture of 20/80 (w/w) at different temperatures. The inset in panel (a) shows the optical images of the bIP aqueous solutions at room temperature and 50 °C, while the inset in panel (d) shows the zeta potential of CNC suspension.

The aqueous solution of the BIP shows a continuous drop in transparency when heated from room temperature to 50 °C. It also exhibited a broad LCST transition (where 10% transmittance decreases as defined during heating) at 37 °C during the heating stage and 34 °C during the cooling stage, as shown in Figure 2a. This measured LCST value, with a 3 °C hysteresis, is slightly higher than but close to the linear PNIPAM, which is 32 °C.^33^ Several studies of branched ionic polymers with PNIPAM macrocations have shown that ionic tethering between branched cores and high density of macrocation tails promote the increase in LCST,^26,34^ while temperature hysteresis is attributed to the difference in diffusion during heating and cooling processes.^18^

Moreover, this thermoresponsive behavior is also reflected in temperature-dependent size change of micellar aggregates, as characterized by DLS in Figures 2b and 2e. The individual bIP micelles exhibited an average size of 227.6 ± 34.9 nm at 25 °C. The size gradually increased to 442.6 ± 15.8 nm at 38 °C, eventually forming much larger aggregates with average size of 613.6 ± 20.6 nm at 50 °C, because of the increased hydrophobicity of terminal PNIPAM chains above LCST. The onset of this transition drives the assembly evolution and aggregation, resulting in larger sizes, broader size distribution, and nanoparticle aggregation, known for branched ionic polymers with PNIPAM branches.^20,39^

Furthermore, zeta-potential measurements confirmed the temperature-induced variation of the surface potential of the ionic polymer micelles (Figure 2c). More specifically, at room temperature (25 °C), the bIP exhibited a surface potential of −10.3 ± 1.0 mV, which gradually shifted to −5.4 ± 1.0 mV at 38 °C and further to 24.1 ± 1.6 mV at 50 °C. As previously reported, hyperbranched polymers bearing sulfuric acid terminates tend to exhibit zeta potential values ranging from −60 to −20 mV, depending on the degree of branching and the hydrophobic–hydrophilic balance.^35,36^ Compared to similar sulfonated HBPs with 50% PNIPAM termination,^26^ the bIP showed a slightly lower surface potential but was closer to carboxylate HBPs with 100% PNIPAM termini.^39^ This suggests that PNIPAM moieties contribute to a greater charge screening effect on the anionic hyperbranched cores. Above LCST, the increasing hydrophobicity of the PNIPAM arms drives a transition from a negative to a positive surface charge state, indicating the collapse of PNIPAM macrocations.^37^

To understand the ongoing changes in suspensions of the mixed CNC and bIPs components, we first examined the size and charge distribution of CNC suspensions, which show the peak hydrodynamic diameter and surface potential to be 119.6 ± 3.2 nm and −52.6 ± 3.9 mV (Figure 2d). For CNC prepared by sulfuric acid hydrolysis, the resulting size distribution and surface potential of our CNC were comparable to those in the literature.^3,38^

When CNCs and bIPs were mixed below LCST, decreases in particle size and shifts in zeta potential were observed as dominated by CNC (see Figures 2e and 2f, as well as Figure S5 in the Supporting Information). Notably, the thermoresponsive behavior of bIPs results in drastic changes in hydrodynamic diameter upon heating over LCST. The average particle size increased from 180.3 ± 13.0 nm below LCST to 666 ± 38.3 nm at 38 °C and 709.8 ± 21.5 nm at 50 °C. These results suggest that the bIP presence enables thermoresponsive behavior of CNC-bIP aggregates.

Similarly, the zeta potential of the mixture also changed as the temperature exceeded the LCST of the bIPs. At RT, the addition of 20 wt % bIPs into the CNC dispersion cause the ζ-potential of the mixture to be −38.7 ± 0.7 mV. Furthermore, as the temperature increased above LCST, ζ-potentials shift to −32.3 ± 0.1 mV and −27.6 ± 0.4 mV for 38 and 50 °C, respectively. While the surface charge of the mixture remains highly influenced by CNCs, the slight shift toward more positive value indicates the intact change of PNIPAM after adsorption of bIP nanoparticles onto the CNC assemblies. This reconfiguration process and interactions during the aggregation stage align with previously observed size changes in hyperbranched polymers with 100% neutralization of PNIPAM chains.^39^

Furthermore, we have conducted additional measurements to validate the long-term stability of the samples (Figure S6 in the Supporting Information). Our additional measurements over different time intervals indicate that significant deviations in size distribution were not observed within the tested time frame of a week. One exception was the bIP solution, which after being stored at room temperature for a week exhibited a relatively broader size distribution while maintaining a peak size of 255 nm (Figure S6c). This may suggest gradual aggregation over prolonged periods, though the samples remained stable over the course of 2 days during film fabrication. Notably, when bIP was adsorbed onto CNC, the mixture demonstrated greater stability, as shown in Figures S6d–S6f. This enhanced stability is primarily attributed to intermolecular ionic interactions which facilitate consistency in the solid layer fabrication.^40^

### Thin-Film Morphology

#### Assembly of Thermoresponsive Hyperbranched Polymers

**Figure 3:**
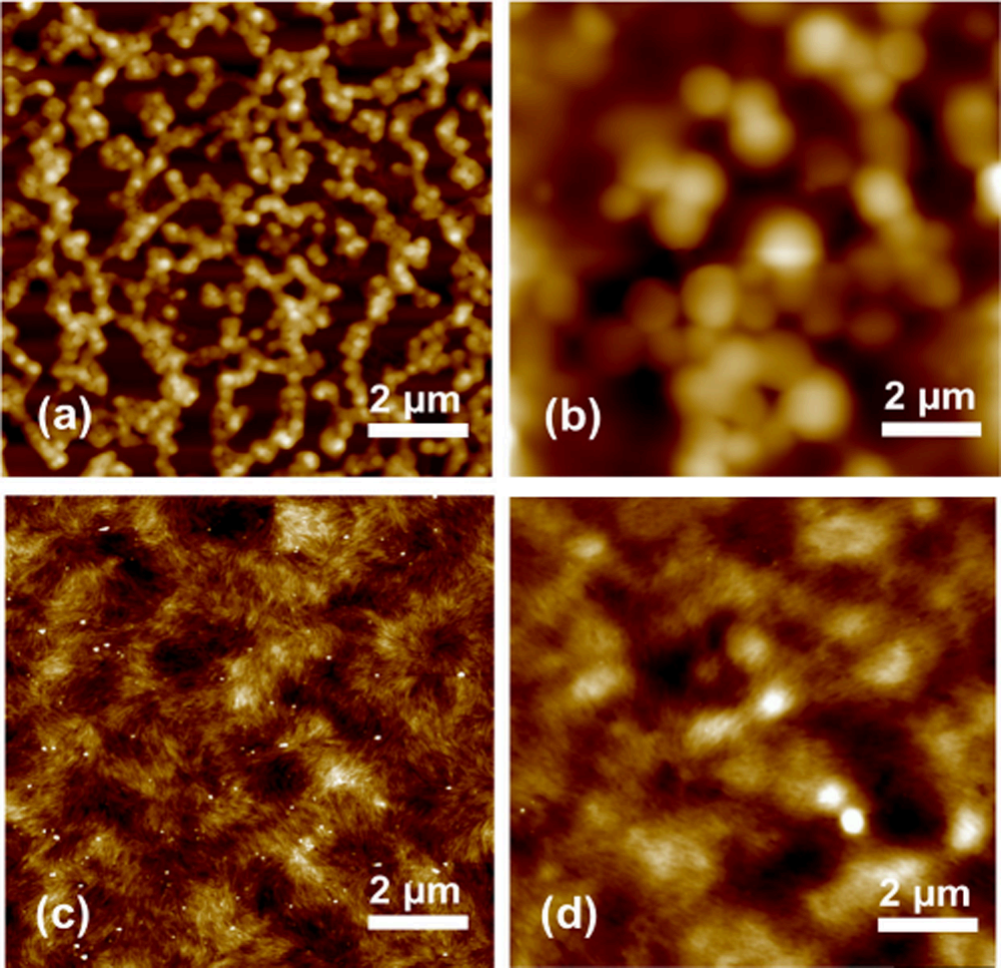
AFM topography images of the bIP thin film surfaces fabricated at (a) *T* = 25 °C and (b) *T* = 50 °C, and thin films of CNC with 20 wt % bIPs at (c) *T* = 25 °C and (d) *T* = 50 °C. Z scales are 200 nm for panel (a), 700 nm for panel (b), 60 nm for panel (c), and 80 nm for panel (d).

Solutions of bIPs were drop-cast on silicon wafers and allowed to dry below and above the LCST temperature (Figures 3a and 3b). The AFM topography images reveal heights of micelles ranging from ∼200 nm to ∼800 nm, resulting from polymer assembly at different drying temperatures. Individual micelles form an interconnected networklike morphology when ionic polymers are dried under ambient conditions, with the average nanoparticle size of 225 ± 21 nm (see Figure S8c in the Supporting Information). In contrast, after annealing at 50 °C, larger micelles appeared as arranged individually, with an average size increasing dramatically to 1170 ± 120 nm (see Figure S8f in the Supporting Information), resulting from core–core fusion of the hydrophobic micelles.^41^

As we demonstrated earlier, due to the ionic nature of the bIPs nanoparticles, PNIPAM macrocationic arms are mobile and capable of hopping between the anionic terminal sites.^26,39^ This dynamic rearrangement leads to the formation of large, mobile coronas surrounded by hydrophobic clusters, which underlies the core–core fusion mechanism. Similar detaching and hopping were also observed in branched ionic polymers with 50%–100% terminated with weakly bound PNIPAM macrocationic arms.^18,39^ Furthermore, along with the increased hydrophobicity originated from the collapse of PNIPAM branches above the LCST transition, the variation of morphologies observed below and above LCST suggest a reconfiguration between the sulfonate hyperbranched cores and amine-terminated PNIPAM arms.^18^

#### Mixed Assemblies of Ionic Polymers and CNCs

The uniform dispersion of bIPs in the assembly of CNCs without any phase separation can be witnessed in Figure 3c. Similar to the pure CNCs, which exhibit locally oriented CNC bundles (see Figure S7 in the Supporting Information), the coassemblies of bIPs and CNCs maintain the anisotropic bundles, with uniformly embedded nanoparticles.

When films were prepared above the LSCT transition of PNIPAM, the coassemblies exhibited increased particle sizes while a decrease in the number of particles, as shown in Figure 3d. The decrease in the total amount of bIP nanoparticles is attributed to the aggregation and reconfiguration of thermoresponsive ionic polymers at the same local concentration. Furthermore, the general chiral nematic organization with helical morphology is retained after treatment at elevated temperatures. This indicates that the assembly of CNCs and branched ionic polymers remains stable, even when PNIPAM chains are collapsed, due to the remaining hydrophilicity of completely terminated bIPs (Figure 3d).

The morphological reconfiguration of bIPs was also inherited when mixed with CNCs, indicating the potential stimuli-responsive behavior of coassembled films. The drop-cast thin films allow us to visualize micellar assembly and investigate the morphological changes caused by mobile intermolecular interactions between cellulose nanocrystals and nanoparticles of ionic polymers. These findings in the thin films highlight the underlying assembly mechanism, which is expected to persist within the composite central layers and facilitate the continuous integration with outer elastomeric layers in the process of fabrication of layered stacks, as is discussed below.

### Morphology and Structural Colors of Elastomeric Stacks

A facile step-by-step fabrication method was then adopted to form the stack of the bIP-CNC nanocomposite layer between two outer layers of PU elastomers, making polymer composite laminates (Scheme 1).

**Figure 4:**
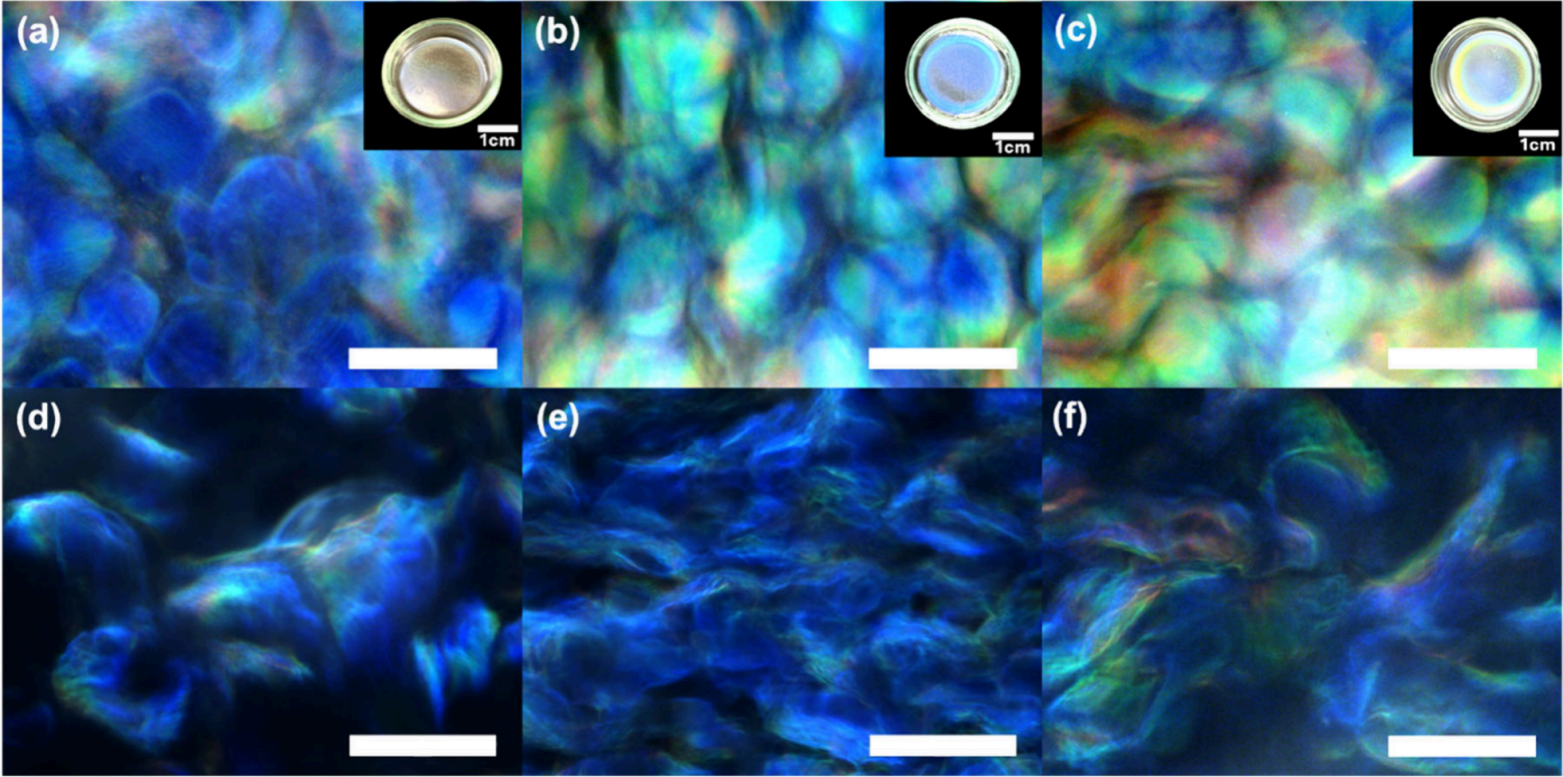
Optical microscopy images with insets showing film appearances of layered composites with different loadings of bIPs (a–c) in bright-field mode and (d–f) in dark-field mode, for pure CNCs (panels (a) and (d)), CNCs with 10 wt % bIPs (panels (b) and (e)), and CNCs with 20 wt % bIPs (panels (c) and (f)). All scale bars are 50 μm unless otherwise specified.

The fabricated layered stacks with the bIPs loading increasing from 0 to 20 wt % were first examined under optical microscopy in the bright field reflection mode (Figures 4a–c). Characteristic tactoid morphology formed by the chiral nematic assembly and the total fingerprint texture of chiral nematic organization were observed in the dark-field optical reflection mode in Figures 4d–f.

It is important to note that all laminates retained their unique structural color of the central layer with vivid iridescence. The blueish color indicates the formation of iridescent CNC films onto the PU substrates.^3^ Moreover, redshifts in color iridescence from blue to green-yellow (as shown in Figures 4 and S9) were also observed for composites with the gradual increase of bIP loading from 10 wt % to 20 wt %, indicating the enlarged pitch length after the coassembly with bIPs and their intercalation into the helical CNC tactoids, usually observed in mixed CNC composites.^5,42^

**Figure 5:**
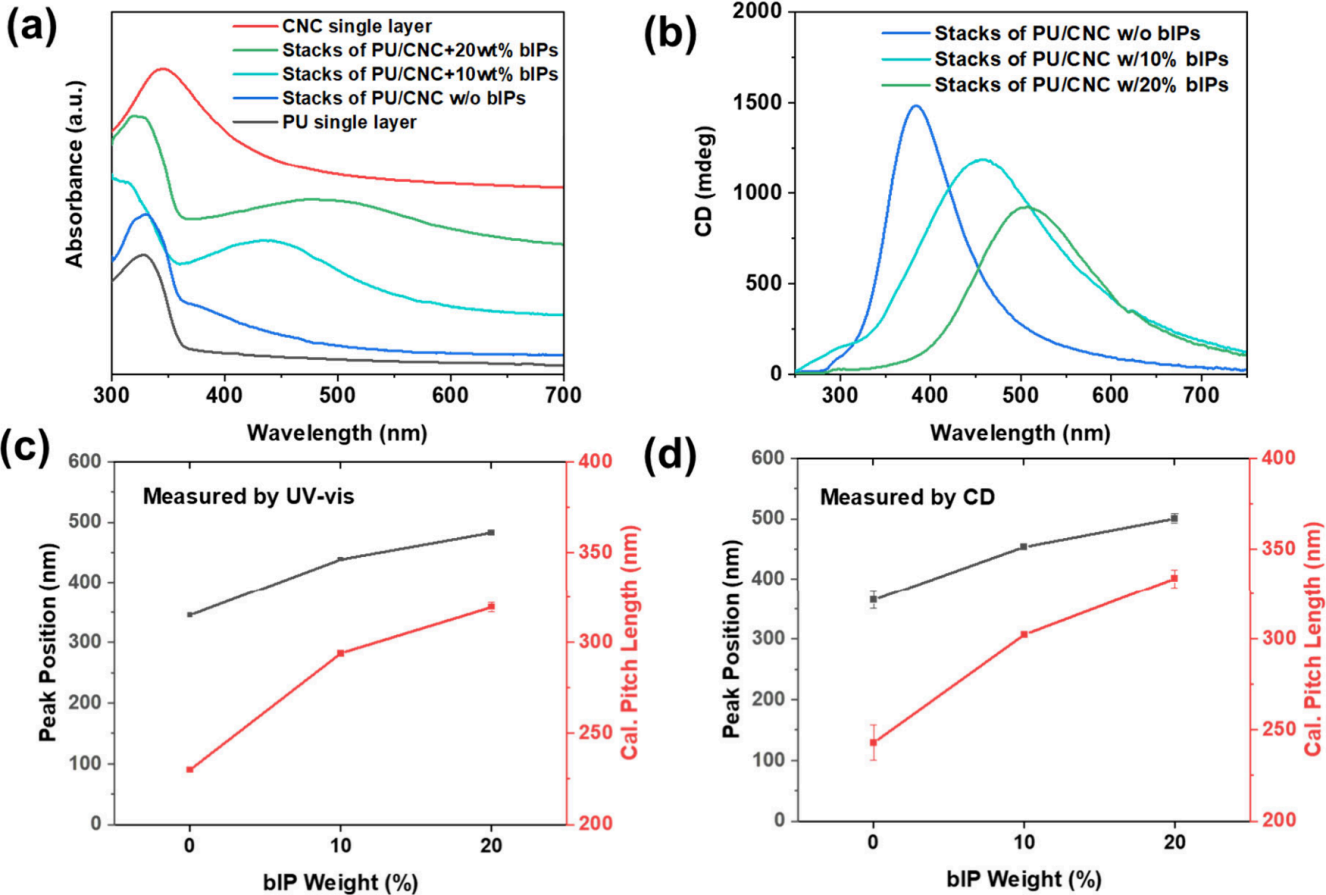
(a) UV–vis absorbance and (b) CD spectra of CNC-PU laminates with different bIP contents; (c, d) corresponding peak position vs pitch length comparison.

The layered composites were further examined by reflectance UV–vis spectroscopy to compare color iridescence as the loading of bIP increases. All bIP-containing stacks displayed a broad peak in the visible light region, along with a smaller peak around 320 nm in the near-ultraviolet region associated with PU layers, as shown in Figure 5a.^43,44^ Notably, a peak with a right shoulder was observed for CNC/PU stacks without bIP addition (the blue curve in Figure 5a), attributed to the overlap of the CNC reflectance peak at 350 nm with the polyurethane-associated UV absorbance (compared by the black curve in Figure 5a).

Additionally, redshifts in the reflectance peak were also observed with the increasing bIP content, shifting from 378 nm to 435 and 487 nm, respectively, for CNC/PU without bIPs, to layered composites with 10 and 20 wt % loading of bIPs, as shown in Figure 5a. A total red shift of 155 nm suggests significant increase in the pitch length of helical organization after intercalation of ionic polymer component. From the peak position, the pitch length *P* was calculated using maximum reflected light wavelength, λ_max_:^45^
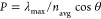
1where θ is the angle between the incident light and the film surface and *n*_avg_ is the average refractive index of the CNC films (*n*_avg_ = 1.5).^45,46^

These calculations show that the average pitch length increased from 252 nm for self-assembled CNC monolayers to 325 nm for composites with 20 wt % bIPs (Figure 5c).

In addition, the light polarization for different laminates was examined by CD to evaluate the reflection of the chiral organization of CNCs influenced by the blend with bIPs. In contrast to pristine CNCs stacked between PU, which revealed the common left-handed peak at 384 nm, both bIP-incorporated CNC layers exhibited redshift to 504 nm for 20 wt % loading (Figure 5b). CD measurements confirmed the consistent left-handed chirality stemming from pristine nanocrystals, which is preserved after the intercalation of ionic polymers into the CNC matrices. Notably, slight peak broadening and decreased intensity was observed in CD spectra, as the full-width half-maximum (fwhm) of the peaks increased from 91 nm for CNC/PU to 147 nm (Figure 5d), suggesting more disperse assembly in the presence of an ionic polymer component.

**Figure 6:**
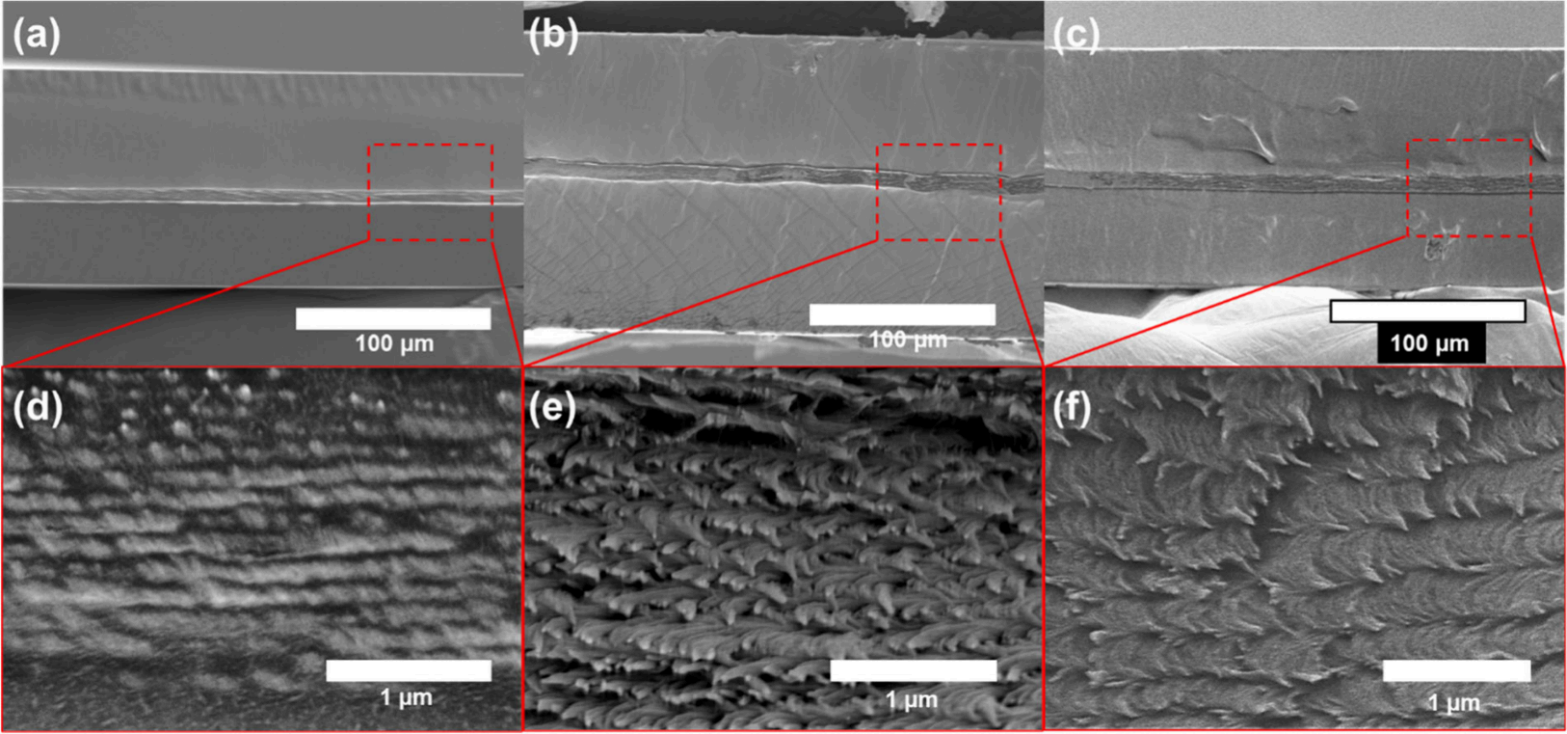
SEM images of stacks showing sandwiched structures from (a) pristine CNCs between PU, (b) CNCs with 10% bIPs between PU, and (c) CNC with 20% bIPs between PU. (d–f) High-resolution images of the Bouligand structures showcasing the helical organization of the central CNC layer.

The morphology of the layered composite film was investigated by SEM imaging of the sample cross sections (Figure 6). The film thickness was measured as 111.6 ± 0.2 μm for stacks with pristine CNC, where the CNC layer was 7.0 ± 0.3 μm.

After adding 10 and 20 wt % bIPs, the CNC layer thickness increased to 8.7 ± 0.7 and 11.3 ± 0.6 μm, respectively. The characteristic periodic Bouligand morphology was observed in central layers at higher magnification, demonstrating that the traditional helical organization with expanded pitch length was preserved under all loading conditions (Figures 6a–c).^47,48^

All layered composite films demonstrated the well-preserved helicoidal organization of CNC layers alternately stacked between the PU layers. An increase in the pitch length follows the same trend deduced from the reflectance peak in the UV–vis spectra (Figure 6d–f). The pitch length increased from 270 ± 10 nm for stacks with pristine CNC to 370 ± 41 and 398 ± 35 nm for CNCs with 10 and 20 wt % bIPs loading, respectively. The measured pitch length values from the SEM were somewhat higher than the calculated values from the reflection peak position in the UV–vis spectra due to distorted/inclined cross-section morphology images.

### Mechanical Performance of the Stacked Laminates

Mechanical tensile tests on laminated composites were performed to understand the influence of obtained multilayered structures on mechanical properties. Pure PU sheets, CNC monolayers, and PU/CNC sandwiched laminates were fabricated for comparison purposes. CNC film shows a brittle fracture behavior with a tensile strength of 40–60 MPa and poor stretchability of 0.5% (Figure S10a in the Supporting Information).

**Figure 7:**
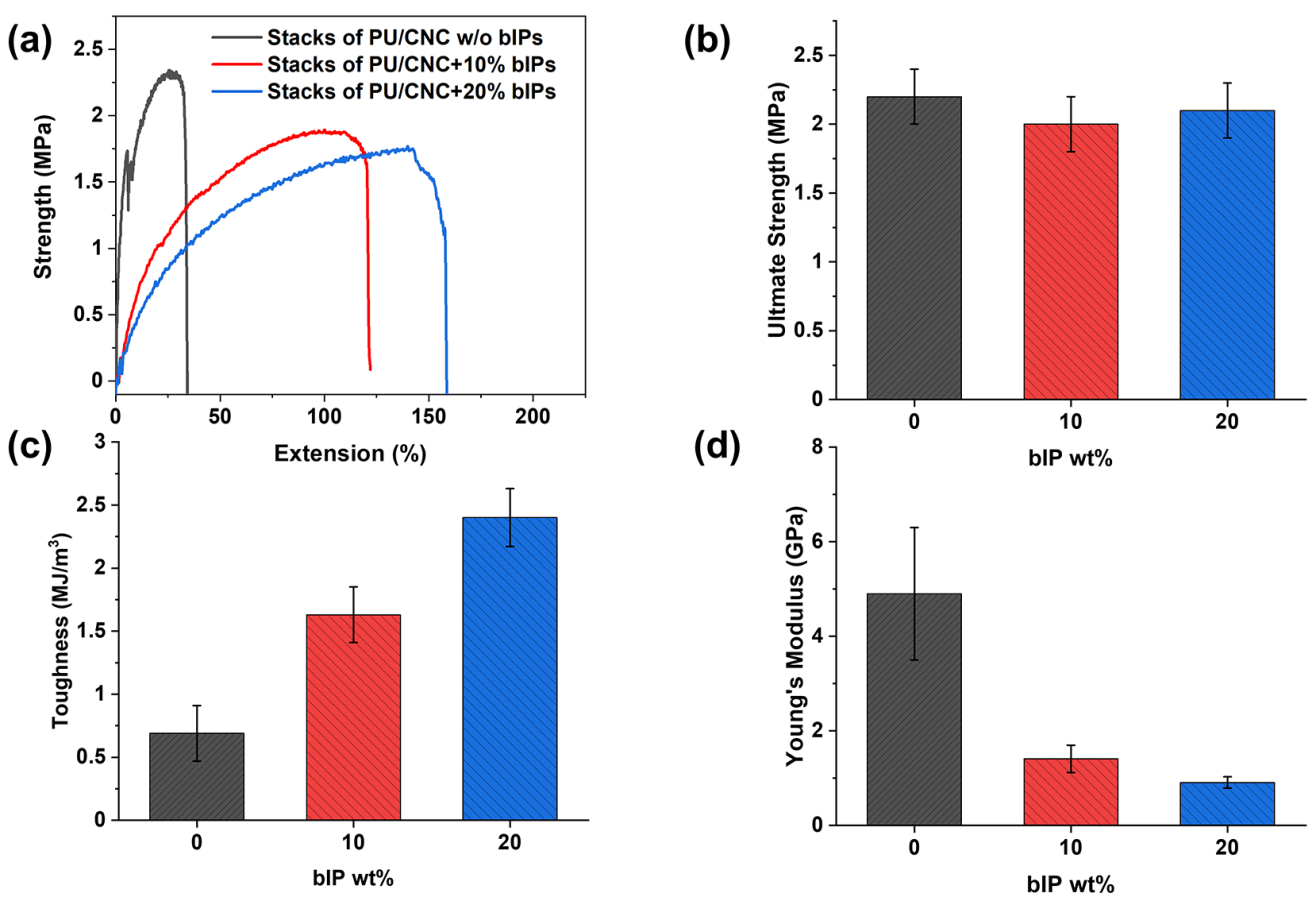
(a) Tensile stress–strain curves for layered stacks with different bIP loading levels and comparison of (b) ultimate strength, (c) toughness, and (d) calculated Young’s modulus for all layered stacks.

At the same time, PU is an elastomeric polymer with optimal extension at break, reaching 250%, as shown in Figure S10b. However, direct mixing these two materials will result in a suppression of iridescent coloration and compromised mechanical stretchability.^49^ In contrast, the elastomeric layered stacks fabricated here showed great improvement in extension at break (30% ± 10%) and ultimate strength (2.2 ± 0.2 MPa) while preserving the vivid colors during the full stretching cycle (Figure 7a).

Furthermore, the intercalation of ionic polymer nanoparticles into the CNC matrix leads to a significant improvement of stretchability with elongation to break of 100% ± 24% and 133% ± 19%, for 10 and 20 wt % loading of bIPs, respectively. Moreover, toughness increases manyfold to 2.4 ± 0.2 MJ/m^3^ at the highest load (see Figure 7c and Table 2). The toughness enhancement was not at the cost of the ultimate strength, which remains approximately at 2 MPa across all samples (Figures 7b and 7c).

**Table 2:** Mechanical Properties of the Layered Composites and Individual Layers

sample	ultimate strength (MPa)	Young’s modulus (GPa)	extension at break (%)	toughness (MJ/m^3^)
pristine CNC films	44.9 ± 5.6	8.0 ± 0.1	0.8 ± 0.2	0.2 ± 0.1
pristine PU films	1.0 ± 0.04	0.14 ± 0.003	230 ± 15	1.6 ± 0.1
PU/CNC/PU stacks	2.2 ± 0.2	4.9 ± 1.4	30 ± 10	0.7 ± 0.2
PU/CNC+10%bIP/PU	2.0 ± 0.2	1.4 ± 0.3	100 ± 24	1.6 ± 0.2
PU/CNC+20%bIP/PU	2.1 ± 0.2	0.9 ± 0.1	133 ± 19	2.4 ± 0.2

Next, the Young’s modulus was calculated and compared for each composition from stress–strain data (Figure 7d). While pure CNC films prepared exhibited a traditionally high elastic modulus of 8.0 ± 0.1 GPa, the PU-sandwiched CNC laminates showed a somewhat reduced elastic modulus of 4.9 ± 1.4 GPa. The introduction of bIPs resulted in further softening to 1.4 ± 0.3 and 0.9 ± 0.1 GPa for 10 and 20 wt % bIP loadings, respectively. Overall, incorporating bIPs induced a typical softening along with notable gains in stretchability and overall toughness of composite layered films in comparison to PU elastomers (see summary in Table 2).

**Figure 8:**
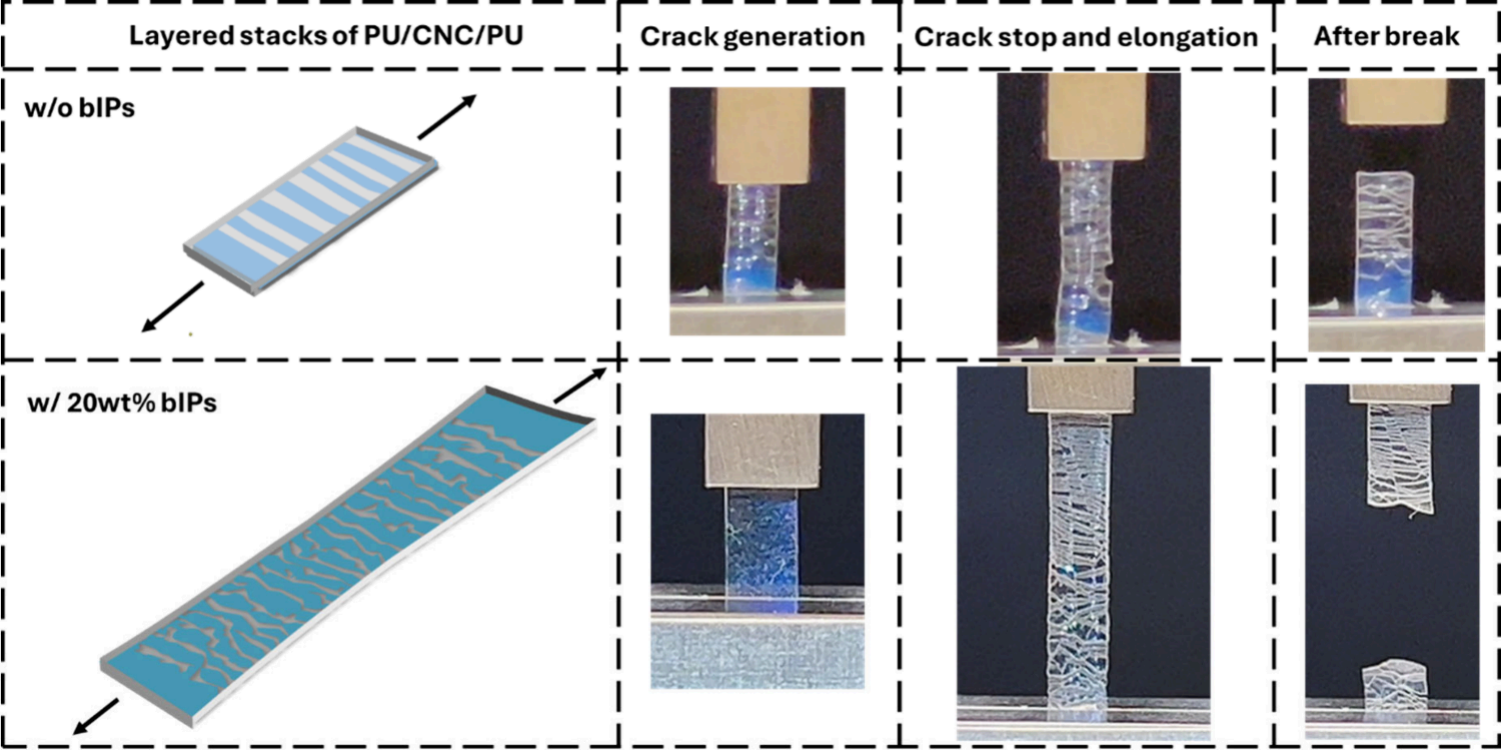
Multiple cracking appearance and propagation in CNC/PU stacks without bIPs (first row) and with 20 wt % bIPs (second row): general schematics and optical images from Video S1.

The deformation and fracture behavior of composite laminates was captured in real-time video during tensile tests (Videos S1). As observed, the deformation unfolded in three stages, including initial crack emerging, followed by crack propagation and stoppings, and formation on new cracks and post-cracking elongation before the final break (Figure 8).

During the initial deformation phase, layered stacks with CNC-bIPs layers exhibited a uniform crack initiation along the sample, reflecting uniform stress transfer facilitated by the softening effect. After initial cracks stopped, the gradual appearance of new numerous horizontal microcracks within the intermediate layer resulted in a dense network of midsized cracks (Figure 8). These small, evenly distributed crack networks efficiently dissipated energy, contributing to superior stretchability of total laminated composites. But the interwoven crack network does not reach the catastrophic stage, with individual cracks basically arrested by crossing bridges between each layer.

In contrast, polymer laminates with a pristine CNC central layer but without an ionic polymer additive exhibited a distinctly different behavior, where the intermediate layer readily fractured into larger, less uniform pieces. The high stiffness of the nanocrystal-only central layer inhibited the formation of smaller, energy-dissipating cracks, leading to earlier fast failure with a fast catastrophic crack propagation across the total width of the sample. Pristine CNC laminates showed fewer, larger cracks propagating rapidly with low chance to arrest crack growth at initial stages (Figure 8).

As the crack network formed at the initial stage, the composite laminates with CNC layers containing bIPs component demonstrated enhanced crack-blunting behavior, characterized by the formation of a unique denser network of bridged and arrested microcracks that resisted fast growth and catastrophic failure. The bIP modified laminates exhibited effective stress redistribution, allowing the formation of interconnected microcrack networks that prevented the propagation of large catastrophic cracks.

Thus, we propose that the critical mechanism for delayed fracture and long extension can be attributed to the synergistic effect of elastomeric PU layers and a malleable CNC-bIP central layer, which interact cohesively across both the nanoscale and molecular levels during the extreme deformation. Co-packing CNCs and bIPs at the nanoscale creates a highly organized ionic network driven by strong ionic interactions. This tightly coupled arrangement leads to a denser and more uniform distribution of numerous partially opened microcracks formed during large deformation.

The dense network prevents stress localization and enables systematic load transfer across the nanocrystal layers, maintaining structural integrity under strain. Moreover, the ionically coupled nanocrystal–nanoparticle organization stabilized the interaction with protecting elastomeric layers, thus facilitating uniform stress distribution across the rage areas. The flexibility and elasticity of elastomeric PU layers facilitate absorbing and dissipating strain energy during elongation. As microcracks within central layers form and propagate, the surrounding elastomeric layers stretch, contributing significantly to the overall composite film extension without a catastrophic break. This dynamic interplay between the ionic coupling within central layer and at interfaces with outmost layers creates a multi-two-sided reinforcement that contribute into crack formation and propagation dynamics. This synergistic reinforcement effect, driven by a network of ionic coupling, represents a significant advancement in designing mechanically robust yet highly stretchable composite films.

### Color Responsive Behavior of Stacked Laminates

Finally, we examined the color-changing ability of our laminates under different relative humidity (RH) by varying the temperature across the LCST in the humid environment.

As known, temperature-induced color change rarely happens for dry solid films because the pitch length of assembled CNC organization is kinetically frozen during the process of EISA and full water evaporation.^50^ For this reason, different types of hygroscopic components were intercalated inside the helical pitch of CNC to mediate the adsorption/desorption of the moisture to achieve macroscopic color change in CNC materials with PNIPAM components.^8,16,51^

**Figure 9:**
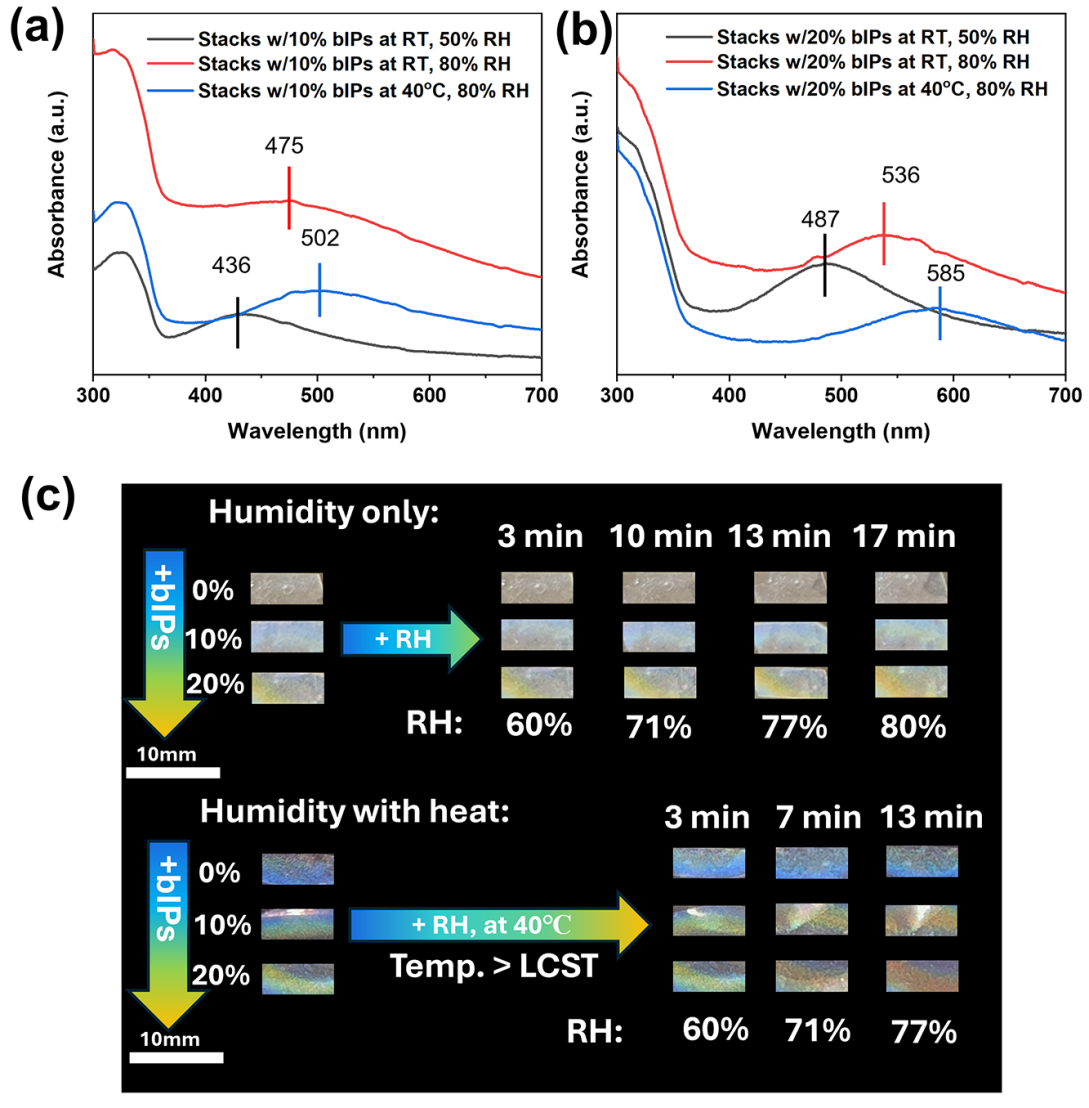
UV–vis spectra of the PU/CNC stacks that contains (a) 10 wt % bIPs and (b) 20 wt % bIPs at different humidity and temperature conditions with (c) corresponding color appearances of laminates cut into strips and put in a humidified incubator with a hygrometer monitoring humidity and temperature with recorded time dependence.

In our study, the films were subjected to temperature and humidity increases, and the reflectance peak was monitored in the UV–vis spectra immediately after treatments (see Figures 9a and 9b). Laminates with both 10% and 20% bIPs were put into a humidified incubator for 17 min, where the RH increased from 50% to 80%. The increased humidity at room temperature resulted in redshifts on UV–vis spectra that indicated the swelling of helical morphology and expansion of pitch (Figures 9a and 9b). The gradual color changes become visible to the naked eye (Figure 9c) as the shifts of 40 and 50 nm appear for the bIP loading level of 10 and 20 wt %, respectively.

Further monitoring of UV peak redshift was conducted to study the impact of temperature on pitch length changes below and above the LCST. It was to note that at the ambient room humidity of RH ≈ 50% no noticeable spectral shifts for all composite laminates. Therefore, we further tested responsive behavior under high humidity conditions, enabling sufficient mobility (RH above 80%) (blue curves in Figures 9a and 9b). After staying in the heated and humidified incubator for 13 min, all laminated films showed noticeable color changes with RH only reaching 77%. The significant redshifts in UV–vis peaks were observed to a total of 76 and 98 nm for 10 and 20 wt % of bIPs content, respectively.

While a higher bIP content in the central layers promotes relatively larger redshifts, the heat annealing further facilitated the water absorption. It may suggest the critical role of the hydrophilic–hydrophobic transition of bIP compounds in water trapping as synergized by the thick outer PU layers. Overall these results indicate that water vapor transport is significantly enhanced in a high humidity environment above the LCST of the bIP compounds.

This humid environment promotes the diffusion of water molecules to infiltrate from the edges of the laminates, particularly where the central layer is exposed by cuts. Subsequently, elevated temperatures provide additional thermal energy to the water vapor, which could be readily absorbed by the hydrophilic components within the helical organization, facilitating to easy intercalation into the pitch.^52,53^ Furthermore, as the hydrophilic PNIPAM moieties undergo a thermoresponsive size increase, the increased moisture content from water vapors could fill interstitial spaces created by the collapsed nanoparticle aggregates above the LCST within the CNC/bIP composite layer, leading to a larger expansion of the pitch length of the cellulose nanocrystal helical assemblies.

The color changing ability was also impacted by the drying process of the CNC/bIP interior layers. Notably, laminates exhibited color changes in a circular pattern across the cut strips to a certain extent. This phenomenon could be attributed to the slight asymmetric particle deposition caused by the Marangoni effect during the drying process for CNC dispersion as reported for CNC suspension themselves.^54,55^ As a result, the shift in color was more pronounced with higher nanoparticle density, compared to that in disordered regions.

## Conclusion

In conclusion, we demonstrated that the composite laminates containing elastomeric layers and central CNC/branched ionic polymer exhibit outstanding mechanical performance with mediated cracking network formation. In contrast to traditional CNC materials, these layered laminates retain iridescent structural colors while responding to external mechanical stresses and environmental stimuli such as humidity and temperature.

We demonstrated that incorporating the ionic polymer component into the central CNC layers sandwiched between outermost elastomeric layers facilitates dramatically increased stretchability and toughness of trilayered composite laminates. The high extension at break reaching 150% with the ultimate strength at 2.0 MPa is facilitated by the extreme stretchability of the central layer. Excessive stretching causes the formation of continuous networks of transversal cracks along the whole length of the films, resulting in manifold improvement in toughness of the laminated composite. Furthermore, the humidity and thermal stimuli of these responsive films result in an unusually large red-shift (∼100 nm) in the reflectance color after thermal treatment at higher humidity.

Overall, integrating responsive branch ionic polymers within the helical organization of cellulose nanocrystal matrices as mediated by elastomeric layers allows for the fabrication of mechanically robust, highly stretchable, and environmentally adaptive materials, making them suitable for a wide range of applications in adaptive thin films for sustainable photonics and sensing applications.

## Supporting Information Available

Additional experimental details, materials and methods, including NMR, TGA, DSC data, AFM topography and phase images, optical images, tensile tests and additional characterizations (PDF)
Video of mechanical tensile testing showing the cracking process (MP4)
