# Supplementary material for: Stretchable Laminates with Tunable Structural Colors from Layered Stacks of Elastomeric, Ionic, and Natural Polymers
Source: ACS Appl Mater Interfaces. 2025 Mar 31;17(14):21830–42. doi: 10.1021/acsami.5c01880 (PMC11986910; doi:10.1021/acsami.5c01880)
Supplement: Supplementary file 1 — am5c01880_si_001.pdf [file am5c01880_si_001.pdf]

Supporting Information for:

Stretchable Laminates with Tunable Structural Colors from Layered Stacks of Elastomeric, Ionic, and Natural Polymers

Yiming Zhang [a], *Paraskevi Flouda* [a,b], *Valeriia Poliukhova* [a], *Alexandr V. Stryutsky* [c], *Valery V. Shevchenko* [c*], *Vladimir V. Tsukruk* [a*]

[a] School of Materials Science and Engineering, Georgia Institute of Technology, Atlanta, Georgia 30332, United States

[b] Department of Chemical and Environmental Engineering, University of Arizona, Tucson, Arizona 85721, United States

[c] Institute of Macromolecular Chemistry of the National Academy of Sciences of Ukraine, Kyiv 02155, Ukraine

* Corresponding authors, e-mails: valpshevchenko@gmail.com and vladimir@mse.gatech.edu

Supplementary video:

Video S1. Recording comparison of tensile tests using layered composites with pristine CNC and with 20% bIPs in CNC layer.

Materials.

Poly(N-isopropylacrylamide) amine terminated (NH_2-PNIPAM) (Sigma-Aldrich, Mn = 2500 g/mol) and 2-sulfobenzoic acid cyclic anhydride (≥95%) were used as received. Hyperbranched aliphatic polyester polyol (Boltorn H30, Perstorp, Mw = 3500 g/mol, hereinafter referred to as HbP-OH) was purified by precipitation of dimethylformamide (DMF) solution in diethyl ether followed by vacuum drying at 25-30 °C for 6 h. An equivalent MW determined by hydroxyl groups via acylation technique is equal to 117 g/equiv. All solvents, including DMF, diethyl ether and ethanol were dried and distilled before use.

Synthesis of hyperbranched oligoester polysulfonic acid HbP-SA. The compound HbP-SA was obtained by exhaustive acylation of HbP-OH by 2-sulfobenzoic acid cyclic anhydride according to our previous study.[1] 1.51 g (13.5 mg-equiv.) of HBP-OH was reacted with 2.49 g (13.5 mg-equiv.) of 2-sulfobenzoic acid cyclic anhydride in 7 ml of DMF at 80ºC under stirring for 8-10 h. The solvent was partially removed under reduced pressure (1-3 mm Hg) and the synthesized product was precipitated to ether with subsequent drying at 40-50 ºC. Then the product was purified by re-precipitation from ethanol to ether and further dried at 40-50 ºC till constant weight. Yield: 3.87 g (97%). The content of acidic groups in the composition of synthesized hyperbranched sulfone derivative at intermediate stage was determined by reverse acid-base titration.[2] SO_3H groups content: determined 25.1%; calculated 26.8%. The obtained compound is transparent brownish viscous liquid soluble in water, alcohols, DMF, DMSO and insoluble in acetonitrile, hexane, and ether.

Synthesis of thermoresponsive branched ionic polymers (bIPs). The synthesis of the compound was performed via neutralization of 0.21 g (0.71 mg/equiv.) of oligoester polysulfonic acid HBP-SA with 1.79 g (0.71 mg/equiv.) of PNIPAM in 5 ml of ethanol. The solvent was partially removed under reduced pressure (1-3 mm Hg) followed by precipitation of the compound to ether and drying at 40-50 °C. The product was purified by reprecipitation from ethanol to ether and further dried at 40-50 °C till constant weight. Yield 1.88 g (94%). The bIP is a pale yellow solid soluble in water, ethanol, THF, chloroform, DMSO, DMF and insoluble in hexane and ether.

FTIR: ν S=O (1000-1096 cm^{-1}), ν C=O amide I (1642 cm^{-1}), ν NHC=O, δ N-H amide II (1545 cm^{-1}), ν C=O of ester groups (1717, 1734 cm^{-1}), δ C-H of CH_2, δ as C-H of CH_3 (1460 cm^{-1}), δ sy C-H of CH_3 (1367, 1387 cm^{-1}), γ C-H of CH_3 (1096-1311 cm^{-1}), ν C-H of CH_2 (2876, 2936, 2974 cm^{-1}), ν ar C-H (3074 cm^{-1}), ν N-H of ammonium (3288 cm^{-1}) and amide groups (3000-3700 cm^{-1}).

^{1}H NMR (DMSO-d_6): 0.80-1.16 (CH(C**H**$_3$)$_2$, CH$_2$C**H**$_3$, C(CH$_2$CO(O))C**H**$_3$, 1104H), 1.25-1.71 (CH(C(O)NH)C**H**$_2$, 323H), 1.80-2.14 (C**H**(C(O)NH)CH$_2$, 180H), 3.84 (C**H**(CH$_3$)$_2$, 183H)) 2.14-4.50 (C**H**$_2$ of HBP core, C**H**(CH$_3$)$_2$, NH$_3^+$C**H**$_2$, 241H), 6.97-8.16 (Ar-**H**, CH(C(O)N**H**)CH$_2$, 191H), 8.39 (N**H**$_3^+$, 8H).

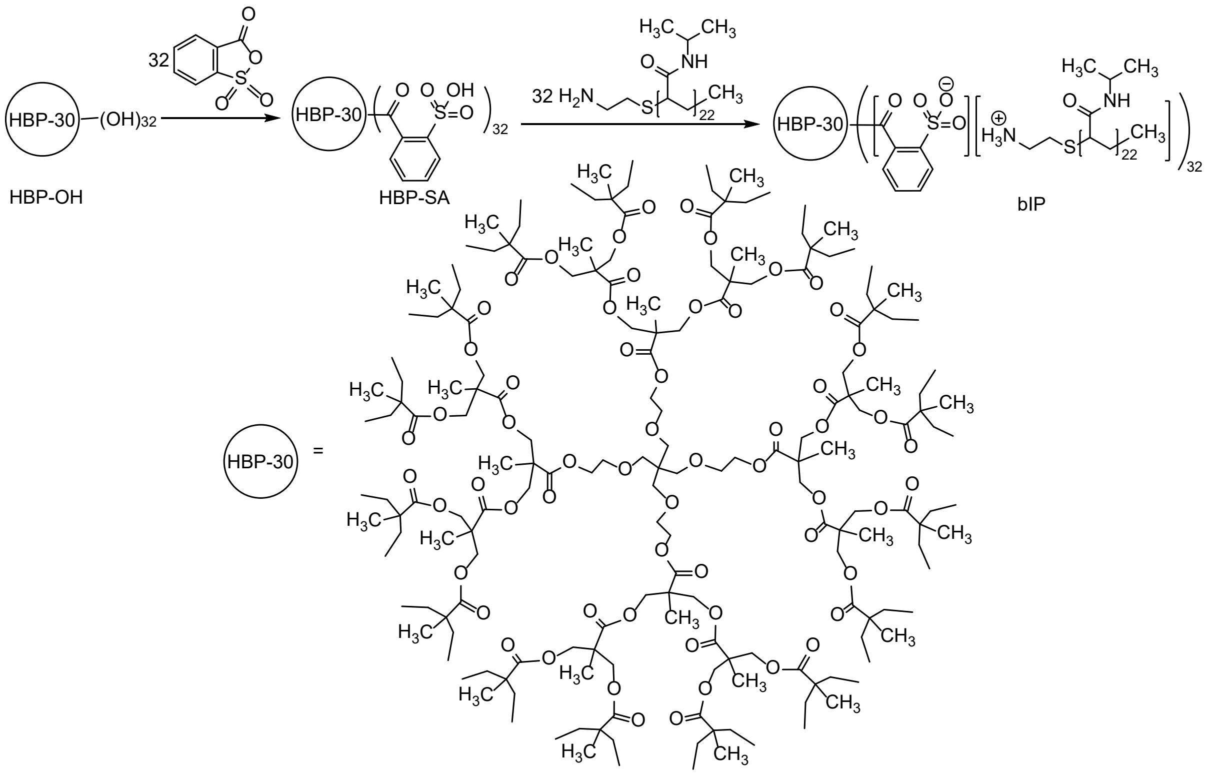

Figure S1. Synthesis and chemical structure of the bIP and hyperbranched core HBP-OH.

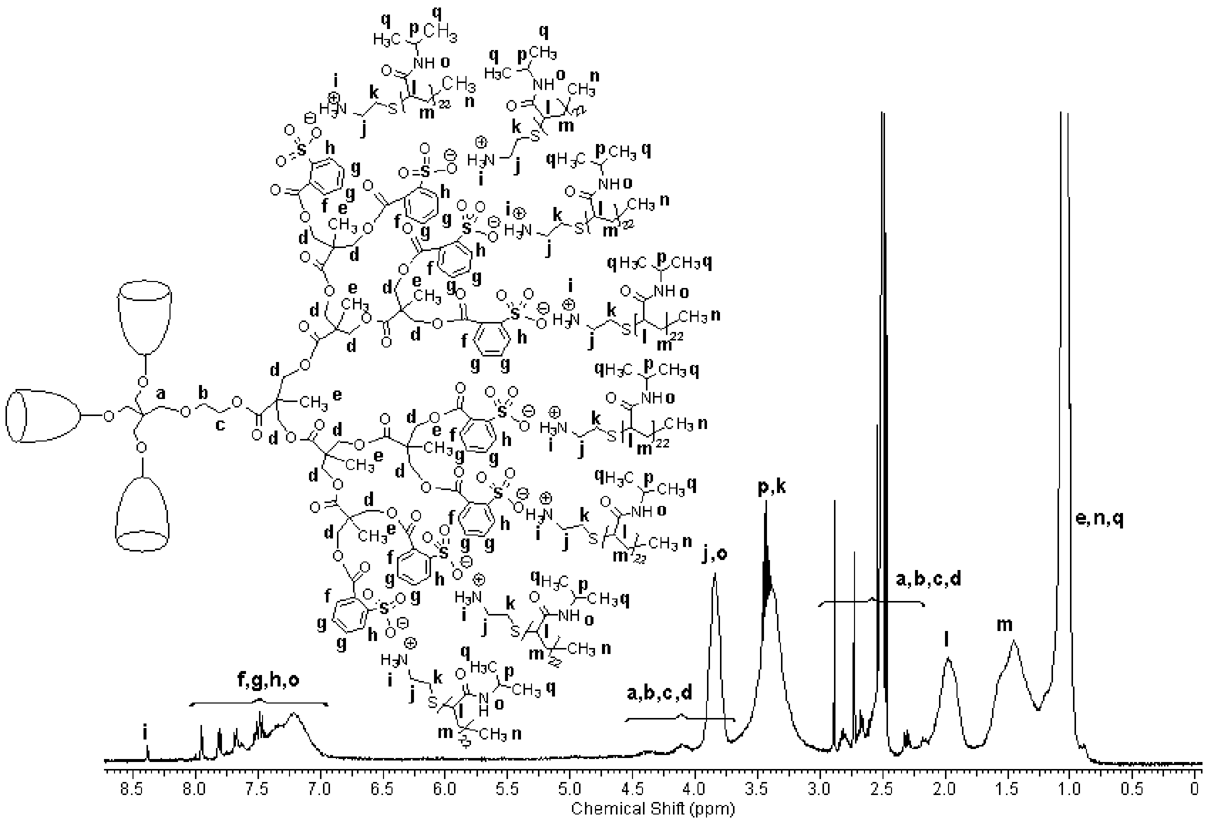

Figure S2. ^{1}H NMR spectrum of the HBP-SA-PNIPAM

Thermal Behavior of the bIP

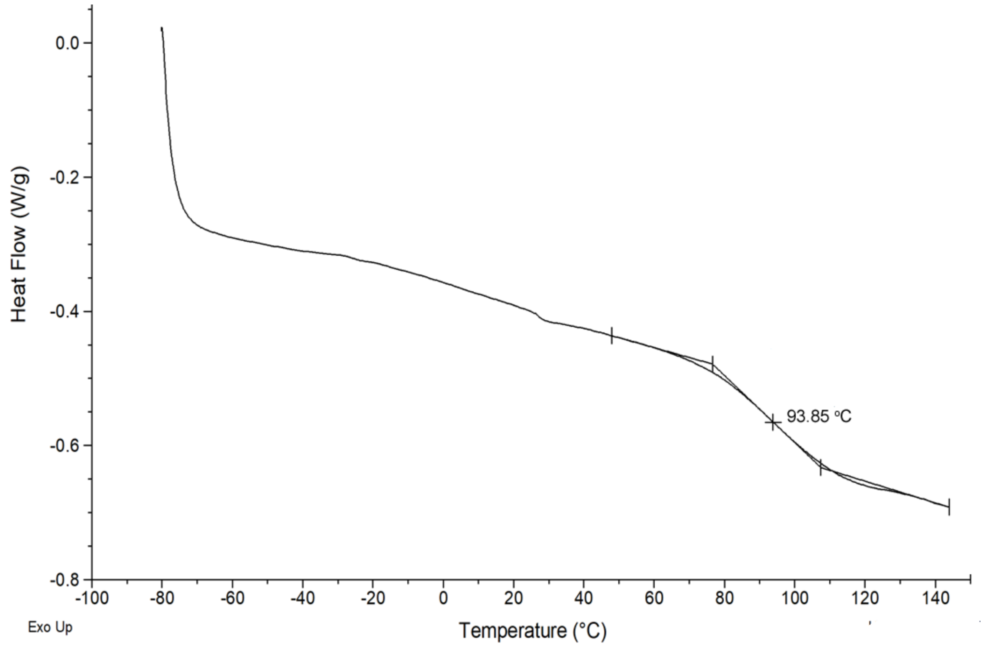

Figure S3. Temperature dependence of heat flow for the synthesized bIP. (2nd scan).

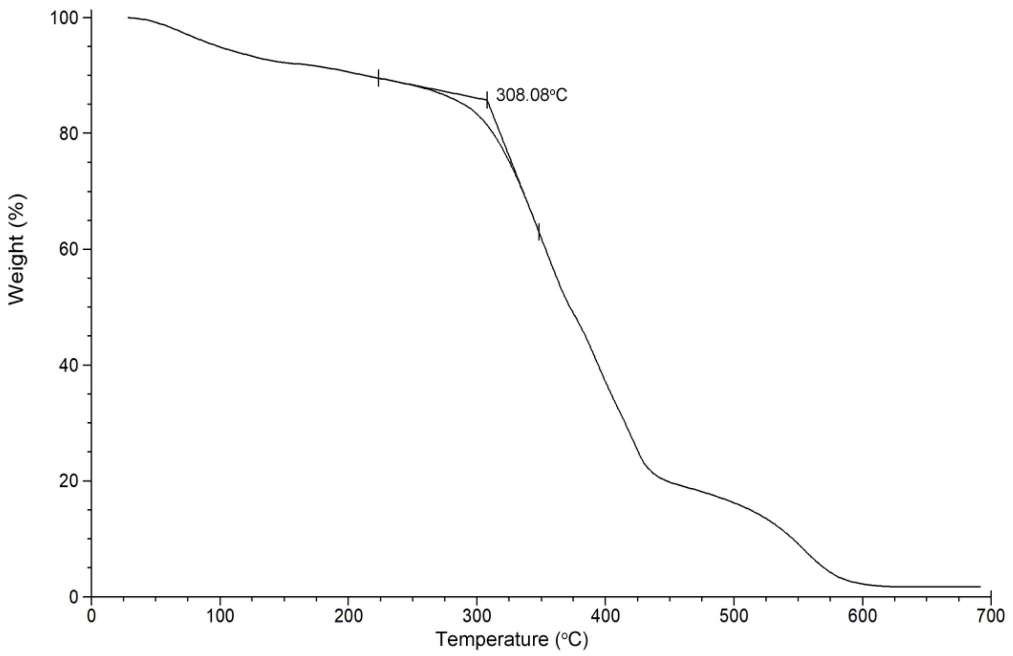

Figure S4. TGA of the synthesized bIP compound.

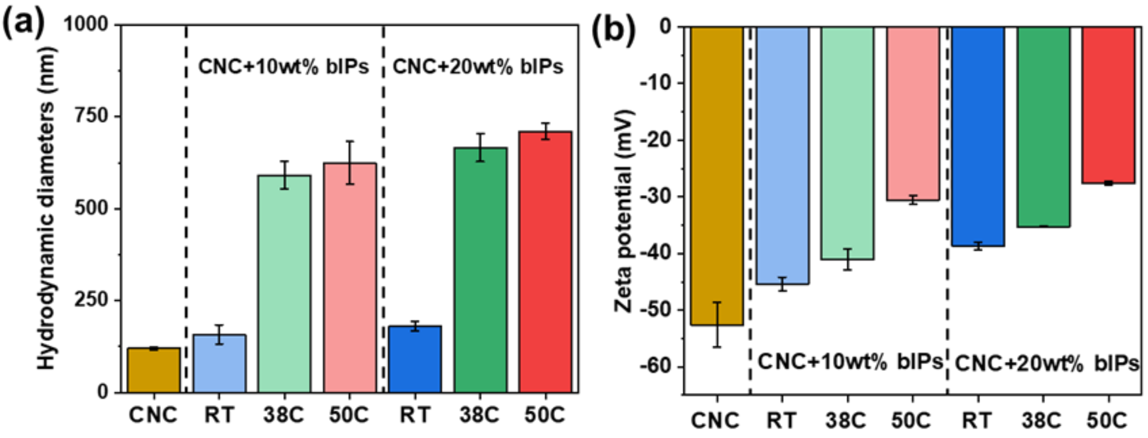

Figure S5. (a) Hydrodynamic diameters and (b) zeta potential for CNC and bIPs mixtures (90:10 and 80:20 w/w) at different temperatures.

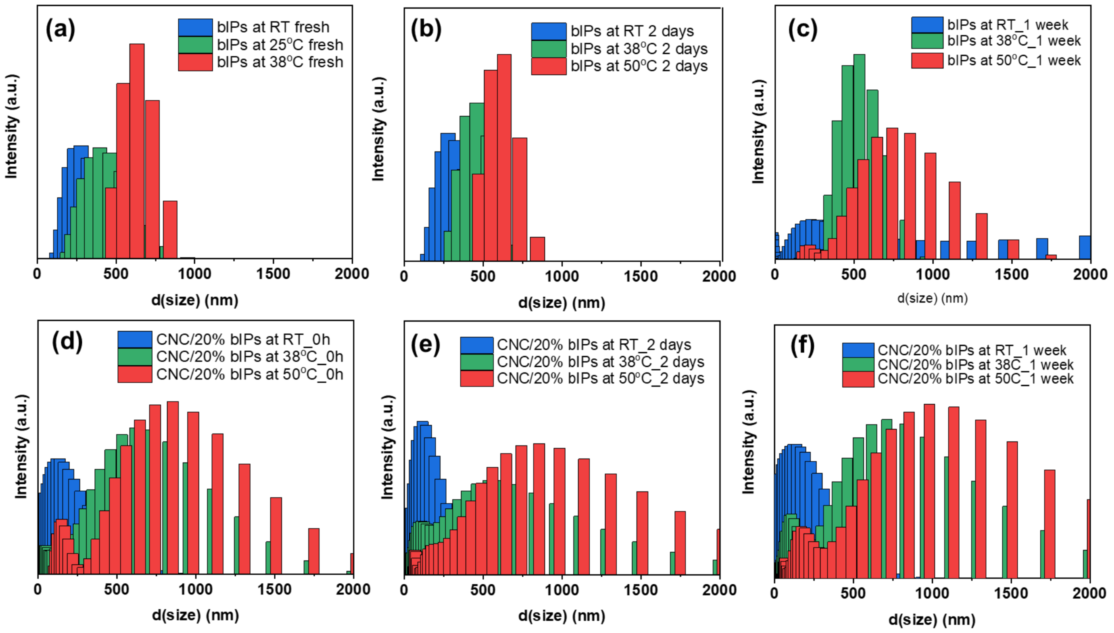

Figure S6. Comparison of size distribution over different time periods measured by DLS. (a-c) bIP and (d-f) mixtures of CNCs and bIPs in 80:20 (w/w) from freshly prepared, to 2 days then to a week.

AFM images of the drop cast samples

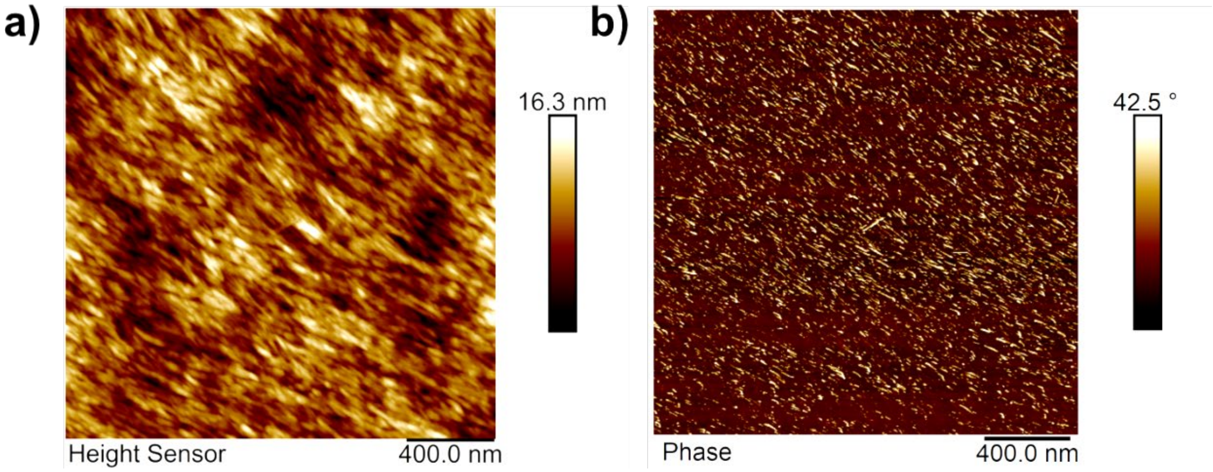

Figure S7. AFM topography (a) and phase images (b) of the CNCs layer.

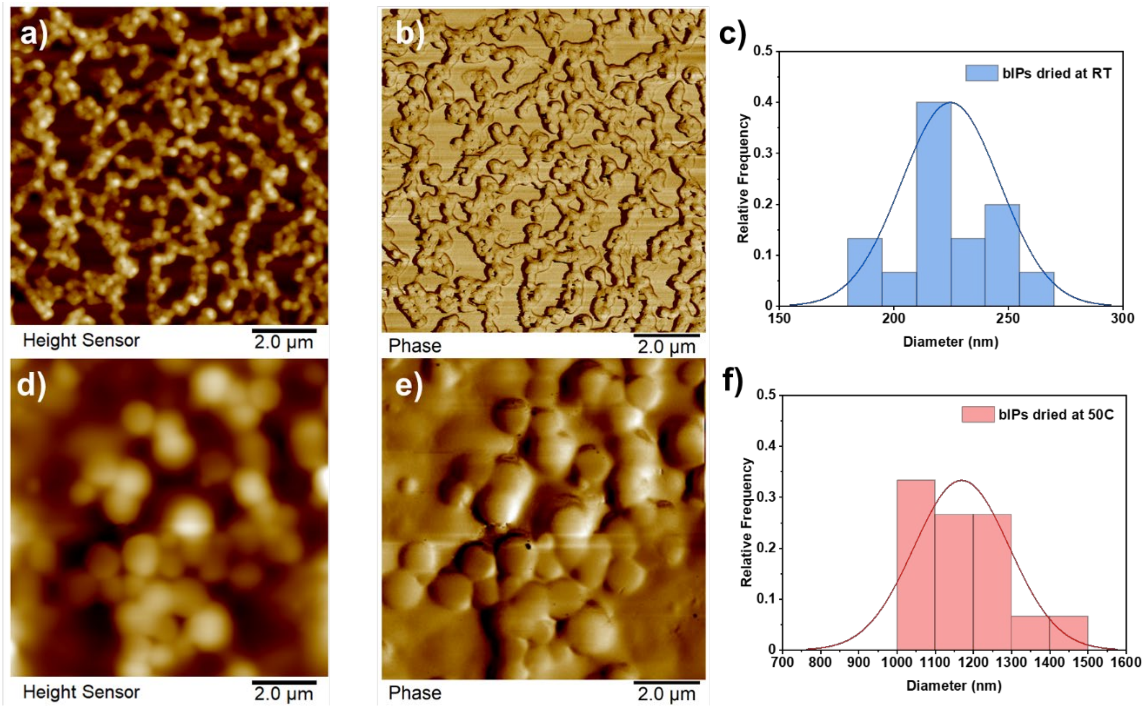

Figure S8. AFM topography and corresponding phase images of bIP nanoparticles (a-c) below and (d-f) above LCST with calculated particle size distribution using ImageJ analysis of AFM images. Z scales are 200 nm for (a), 100º for (b), 700 nm for (d), and 20º for (d).

Optical Microscopy Images.

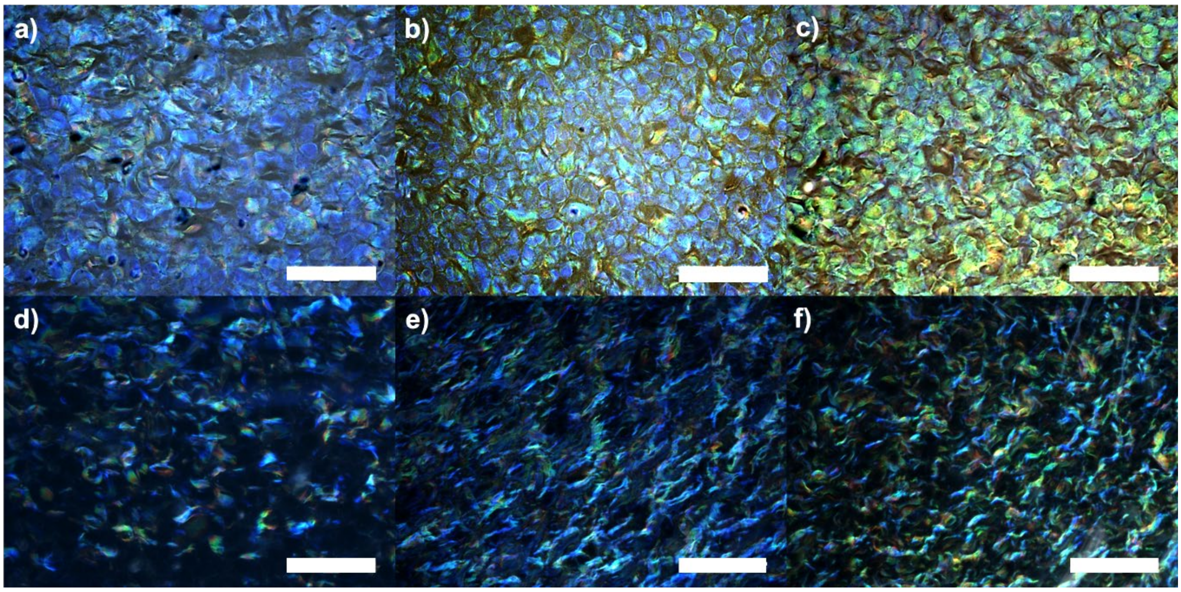

Figure S9. Optical microscopy images of laminates with pristine CNC (a,d), laminates with CNC+10 wt% bIPs (b,e), and laminates with CNC+ 20 wt% bIPs (c,f). All images have the same scale bar of 200 μm.

Mechanical Properties of Individual Components

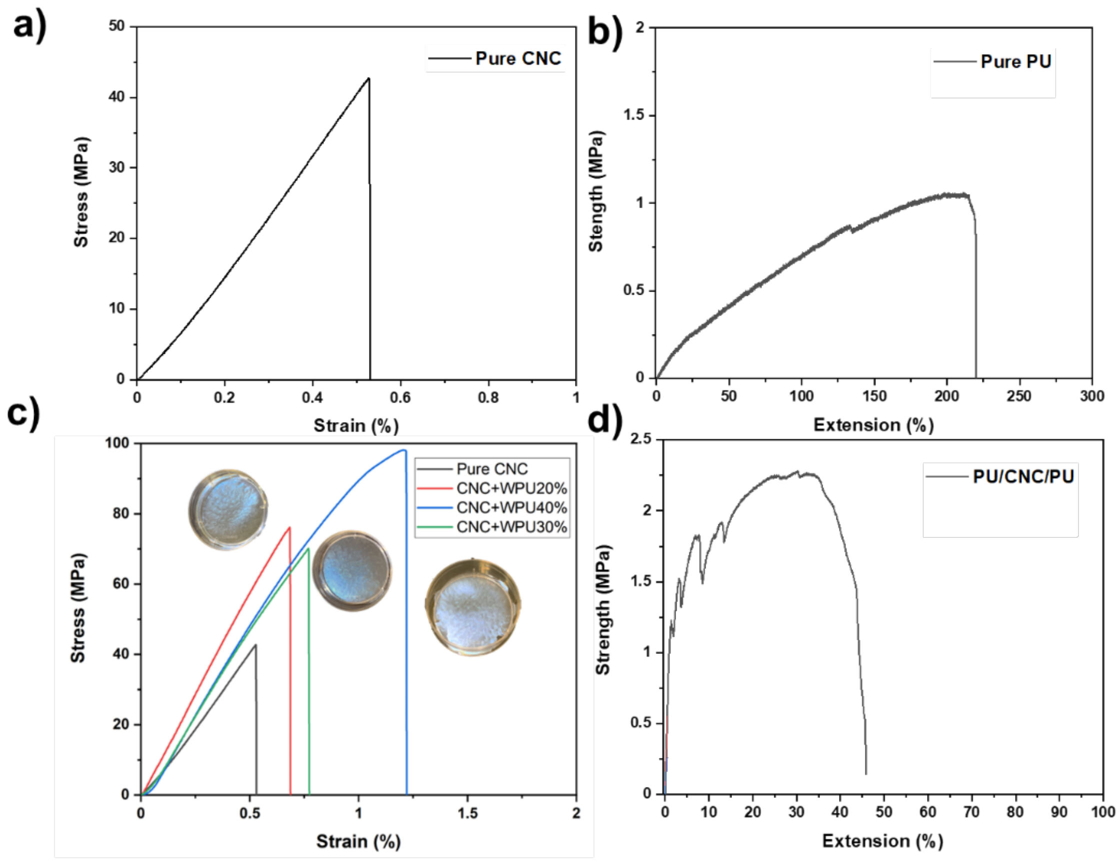

Figure S10. Tensile stress-strain curves of (a) CNC single layers (b) PU (c) direct mixture of CNC and PU with inserts showing film appearances and (d) the layered stacks between PU and CNC.

References

[1] Shevchenko, V. V.; Stryutsky, A. V.; Klymenko, N. S.; Gumenna, M. A.; Fomenko, A. A.; Bliznyuk, V. N.; Trachevsky, V. V.; Davydenko, V. V.; Tsukruk, V. V. Protic and Aprotic Anionic Oligomeric Ionic Liquids. *Polymer* **2014**, *55* (16), 3349–3359. https://doi.org/10.1016/j.polymer.2014.04.020.

[2] Kim, D.; Park, H.; Rhim, J.; Lee, Y. Proton Conductivity and Methanol Transport Behavior of Cross-Linked PVA/PAA/Silica Hybrid Membranes. *Solid State Ion.* **2005**, *176* (1–2), 117–126. https://doi.org/10.1016/j.ssi.2004.07.011.
